# Ultra-Low-Power, Temperature Field-Based Condition Monitoring of Particle Contamination and Overheating in Plain Bearings

**DOI:** 10.3390/s26217011

**Published:** 2026-11-05

**Authors:** Thao Baszenski, Jan-Christoph Möller, Karl-Heinz Kratz, Georg Jacobs, Tobias Gemmeke, Anuj Khare, Benjamin Lehmann, Mattheüs Lucassen

**Affiliations:** 1 Institute for Machine Elements and Systems Engineering (MSE), RWTH Aachen University, 52062 Aachen, Germany; 2 Chair of Integrated Digital Systems and Circuit Design (IDS), RWTH Aachen University, 52074 Aachen, Germany

**Keywords:** plain bearings, sliding bearings, hydrodynamics, condition monitoring, sensor integration, sensor-integrating machine elements, tribology, digitalization, machine elements

## Abstract

Hydrodynamic plain bearings are widely used in heavy-duty applications, e.g., wind turbine drivetrains or ship propulsion systems. However, abnormal events such as mechanical overload causing overheating or particle contamination can lead to rapid bearing damage and the failure of the entire drivetrain. Most existing condition monitoring systems (CMS) cannot detect particle contamination and mechanical overloading in a timely manner or are associated with high costs and installation effort that limit their practical applicability. In this paper, temperature field measurement (TFM) is investigated as a promising approach to address this gap via the development of a sensor-integrating plain bearing (SiPB) prototype. The approach is characterized by its low cost and low energy demand, with the potential for autarkic operation through the thermoelectric harvesting of the frictional waste heat within the bearing. In both overheating tests, characteristic signal discontinuities in the temperature field occurred at least 10 min before the friction torque limit was reached, which served as the criterion for terminating the experiment. Particle contamination resulted in a pronounced increase in the scattering in the temperature field over the entire duration of the experiment compared to regular operation. The interquartile range (IQR) of the mean temperature was identified as a promising characteristic; in the single-contamination test, the IQR was increased by a factor of 2.6 compared with regular operation. These characteristics were derived descriptively from one test per abnormal event; repeated tests are required to validate them. The energy demand of the SiPB prototype is determined as a function of the sampling rate (1.3 mW at a sampling rate of 0.2 Hz). The temperature differences required for cold start and the supply mode of the DC-DC converter necessary for supplying the required operating voltage of the SiPB prototype via thermoelectric energy harvesting are determined (approximately 2 and 5 K, respectively). Based on simulation data, this corresponds to the magnitude of temperature differences expected in the plain bearing during normal operation, meaning that the autarkic operation of the SiPB can be enabled in the future.

## 1. Introduction

Hydrodynamic radial plain bearings are used in applications not covered by established design standards. An established guideline for the safe design of hydrodynamic, metallic plain bearings is the DIN 31652 standard [1,2,3]. According to this standard, long-term operational safety is defined as the exclusive presence of fluid friction within the plain bearing. The standard does not account for prolonged or frequent operation in the mixed-friction region. Some bearing applications regularly pass through the mixed-friction region as part of standard operation. Examples include start-up and shutdown processes in the automotive sector [4], as well as ramp-up, idle, and emergency shutdown phases during wind turbine operation [5]. For short-term start-up and shutdown processes, DIN standard 31652-3 [3] specifies an empirical, material-independent limit value for the permissible specific pressure (${\bar{p}}_{max}$ = 2.5 MPa), which is, however, chosen very conservatively. The literature indicates that plain bearings can withstand significantly higher loads during start-up and shutdown processes without damage over the long term [6,7,8,9]. Furthermore, in the automotive sector, specific pressures occur in the plain bearings of internal combustion engines during normal operation that far exceed the permissible limit for material safety in plain bearing design according to DIN 31652. For example, in the big-end bearing of the connecting rod, specific pressures of $\bar{p}$ = 100 MPa and higher occur [6,10]. The permanently permissible specific pressure for steady-state operating conditions according to DIN 31652 is only ${\bar{p}}_{limit}$ = 7 MPa. Consequently, the operation of hydrodynamic plain bearings in automotive or wind turbine applications would therefore not be permissible if designed according to the DIN standard. Nevertheless, bearings have been successfully used in series production for several years in wind turbines and for decades in automotive applications. These applications demonstrate that plain bearings can tolerate occasional operation in the mixed-friction regime, even though this is not accounted for by current design standards. If a bearing is operated too frequently or too deeply within the mixed-friction regime, however, the risk of damage increases significantly [11]. Since no standard currently exists for designing plain bearings for deliberate mixed-friction operation, condition monitoring systems (CMS) are employed instead to monitor the frequency and criticality of such mixed-friction events. In addition, particularly severe mixed-friction events can lead to rapid bearing failure. These critical events are abnormal in nature and can, for example, be caused by mechanical overload, which induces severe mixed friction, resulting in excessive heat generation and subsequent failure. Another example is particle contamination, where three-body abrasion caused by the ingressed particles leads to mixed friction [12,13]. At high particle concentrations, this can result in mixed friction over a large contact area, likewise leading to excessive heat generation and failure.

Focused condition monitoring enables the reliable and early detection of emerging damage to machine elements. For hydrodynamic radial plain bearings, a variety of condition monitoring methods, such as lubricant outlet temperature, vibration, or oil condition monitoring, are well established [14]. For the measurement principles mentioned, the corresponding sensors are placed outside the plain bearing. The increased signal transmission path can lead to reduced data quality due to interfering influences. An option for improving measurement quality is the sensor’s integration into the machine element (“in situ”). By minimizing the transmission path of the measurement signal, the sensitivity for detecting changes in the machine element’s condition is maximized. Measurement principles suitable for the in situ monitoring of plain bearings include high-frequency acoustic methods (acoustic emission [15], ultrasound [16]) or inductive displacement sensors for determining the lubrication gap height [14]. However, the mentioned monitoring methods are associated with high costs and/or complex data analysis, as well as high energy consumption. Furthermore, the available CMS for plain bearings require external wiring for data transmission and power supply [17,18]. In particular, in multi-axis rotating systems, such as planetary gears, cable routing is very complex.

Temperature-based condition monitoring is the most frequently used monitoring method for plain bearings [19]. Furthermore, the temperature is an established parameter in the design of plain bearings for evaluating operational safety. Laubichler et al. [20] measured crankshaft main bearing temperatures with thermocouples embedded in all seven main bearing shells to validate a data-driven reference model that predicted the expected temperature based on the engine operating parameters. The goal of the model was to estimate the temperature safety for the motor’s steady-state operating points. In ref. [21], an integrated condition monitoring system for plain bearings in internal combustion engines was developed, which, among other aspects, incorporated printed temperature sensors into the plain bearing running surface. Moreover, DIN 31652-3 specifies empirically based temperature limits for assessing the temperature safety of the bearing during steady-state operation [1]. Since DIN 31652-3 only covers the design for steady-state operation, the oil outlet temperature is defined here as a suitable monitoring parameter. Transient, damage-critical special-case events, however, are not covered by the standard.

Temperature field measurement (TFM) represents a novel monitoring approach in which the temperature field near the running surface of the plain bearing is measured with a high spatial resolution. Originally, this approach was developed to determine the lubrication gap height based on the temperature field within the bearing under steady-state conditions. However, further investigations have shown that this in situ monitoring approach is highly suitable for detecting changing friction conditions in plain bearings [22]. In addition, the developed TFM-based approach relies on cost-effective and commercially available microelectronic components, which could enable widespread field application in the future. Furthermore, the microelectronic components used are energy-efficient to such an extent that their energy requirements can, in principle, be met through energy harvesting, eliminating the need for an external power supply. The feasibility of the TFM approach for the early detection of damage-inducing abnormal events, namely overheating due to overloading and particle contamination, has not yet been investigated. Accordingly, this paper, for the first time, demonstrates that the TFM approach is capable of detecting the failure-causing abnormal events of overloading and particle contamination at an early stage, before they cause damage to the bearing. Section 2.1.1 provides an overview of the authors’ own preliminary work to date regarding the development of TFM-based condition monitoring. In order to ensure that these two abnormal events can be detected in a timely and self-sustaining manner in the future, the energy requirements of the SiPB prototype are determined based on the measurement strategy. Subsequently, the temperature difference required to enable the cold start of thermoelectric energy harvesting will be established.

## 2. Materials and Methods

This section is divided into two parts. Section 2.1 explains the underlying concept of temperature field measurement and the physical principles of thermoelectric energy harvesting. Section 2.2 describes the methodological approach: the prototype of the sensor-integrating plain bearing (SiPB) with the microelectronic components used and the test-bench setup in which the CMS concept is validated for particle contamination and overheating due to mechanical overload. In addition, Section 2.2.3 defines the underlying principles of data analysis, with TFM-based characteristics for detecting overheating and particle contamination discussed in Section 3.

### 2.1. Fundamentals

#### 2.1.1. Temperature Field Measurement

The idea of determining the lubrication gap height based on the temperature field of the plain bearing is rooted in the Gümbel curve. The Gümbel curve is part of DIN standard 31652-2 [2], which provides design guidelines for hydrodynamic radial plain bearings. Since the Gümbel curve is valid only for steady-state operating conditions and its validity is based on the assumption of ideally smooth surfaces, it is not suitable for detecting mixed friction. However, if the lubricant film height falls below the threshold value $h_{lim}$, which marks the transition to the mixed-friction region, the resulting mixed friction causes a local temperature rise at the roughness peaks in contact with each other. A spatially resolved temperature field measurement (TFM) could therefore be suitable for rapidly detecting the transition from the fluid friction region to the mixed-friction region [22]. However, to ensure the maximum sensitivity of the temperature sensor to changing friction conditions in the plain bearing, it is essential to ensure that the thermal connection between the temperature sensor and the plain bearing is optimized. Since temperature sensors should not be directly integrated into the lubrication gap, in accordance with DIN 31692-2 [23], the distance between the friction phenomenon and the temperature sensor must also be minimized. Incipient damage to the plain bearing is accompanied by high heat input into the bearing and could be detected by selecting appropriate temperature-based thresholds via in situ TFM. The sensors are therefore arranged only in the angular range of $180^{\circ }$–$266.25^{\circ }$, which corresponds to the displacement angle range of the shaft in steady-state operation and thus to the measurement field relevant for the Gümbel curve. This range was determined by TEHD simulations in [18]. In [22], the hypothesis was validated with a predecessor prototype with sensors in the lower bearing half ($90^{\circ }$–$270^{\circ }$). Whether rapidly progressing bearing damage caused by abnormal events can be detected early using TFM has so far only been examined simulatively in [22]. Experimental verification has not yet been conducted.

#### 2.1.2. Thermoelectrical Energy Harvesting

The conversion of thermal energy into electrical energy within a thermoelectric generator (TEG) is driven by a dynamic, coupled relationship between three primary thermodynamic and transport phenomena. The fundamental driving force of this system is the Seebeck effect. When a spatial temperature difference, defined as $\Delta T=T_{\mathrm{hot}}-T_{\mathrm{cold}}$, is maintained across the device, charge carriers diffuse from the high-temperature junction toward the low-temperature junction. This migration and subsequent accumulation of charge establishes an open-circuit voltage proportional to the applied temperature difference, expressed as $V_{\mathrm{oc}}=\alpha \cdot \Delta T$, where α represents the total Seebeck coefficient of the thermocouple [24].

Once an external electrical load, $R_{\mathrm{load}}$, is connected to close the circuit, a current, *I*, begins to flow through the matrix, immediately activating two internal electrothermal feedback loops that alter the system’s performance. The first of these phenomena is the Joule heating effect. As the electrical current flows through the inherent internal resistance, $R_{\mathrm{in}}$, of the semiconductor material, it causes internal ohmic heat dissipation equal to $I^{2}R_{\mathrm{in}}$. This internal loss irreversibly reduces the net electrical power output delivered to the external load, which is quantified by the balance $P_{\mathrm{out}}=I\cdot V_{\mathrm{oc}}-I^{2}R_{\mathrm{in}}$ [24]. Simultaneously, the flowing current triggers the Peltier effect, which transports heat from the hot junction to the cold junction at a rate defined by ${\dot{Q}}_{\mathrm{Peltier}}=\alpha \cdot I\cdot T_{\mathrm{hot}}$, effectively decreasing the temperature difference ΔT. According to Bancik et al. [25], a $40\times 44.7\;{\mathrm{mm}}^{2}$ TEG operating at a temperature difference of ΔT=3K yields an output voltage of 44.7mV and harvested power of 1.26mW. Below this temperature difference, the power output of the TEG decreases to 0.194mW for ΔT=2K, while, for larger differences, it rapidly increases (e.g., 2.8mW for ΔT=4K). This makes it suitable for a low-power device operating in the sub-milliwatt to low-milliwatt range.

**Figure 1:**
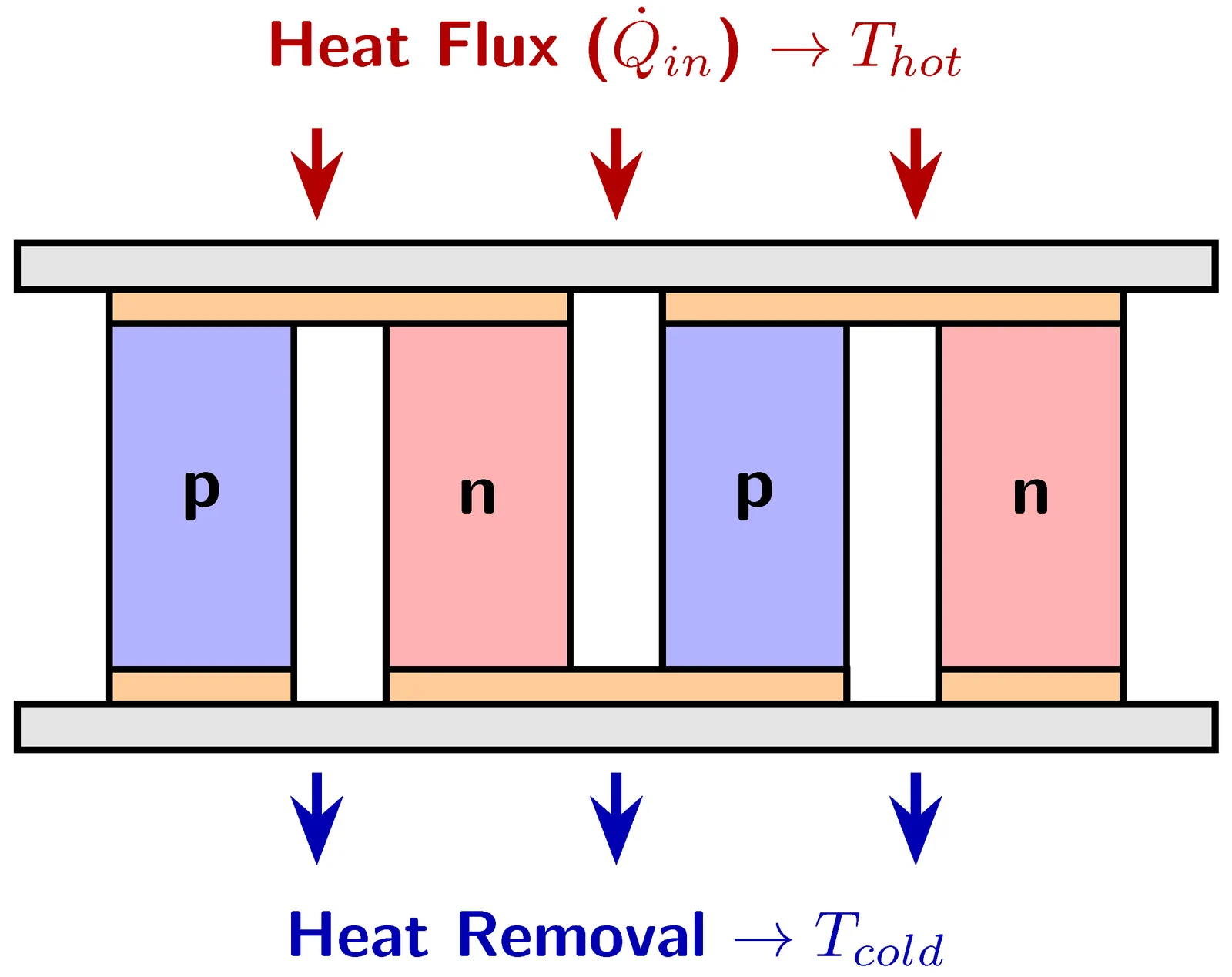
Schematic structure of a multi-couple thermoelectric generator showcasing alternating p-type and n-type semiconductor legs connected electrically in series and thermally in parallel between ceramic substrates.

Figure 1 illustrates the internal structure of a TEG module, which utilizes an alternating π-type architecture where the semiconductor legs are connected electrically in series to maximize the voltage output and thermally in parallel between two ceramic substrates [24].

The voltage generated by the TEGs is too low to power the SiBP directly. The most efficient way to up-convert a voltage is a DC-DC boost converter. The load, however, is dynamic, and a dynamic load may temporarily draw more power than the TEGs can provide. The AEM13920 PMIC combines a DC-DC boost converter on the primary side with a DC-DC buck converter on the secondary side. The boost converts the low TEG voltage into a higher voltage and fills a storage capacitor that is connected between boost and buck. The buck converter converts the high capacitor voltage into a lower output voltage [26]. This architecture decouples the TEGs from the load and enables the matching of the input impedance to the internal resistance of the TEGs for maximum power extraction.

The AEM13920 requires a minimum of 275 mV for cold start [26]. During normal operation, it can harvest starting from 120 mV. The output voltage of a TEG is proportional to the temperature difference. This makes cold start the most critical phase for low-ΔT applications.

### 2.2. Methodology

#### 2.2.1. Sensor-Integrating Plain Bearing

**Figure 2:**
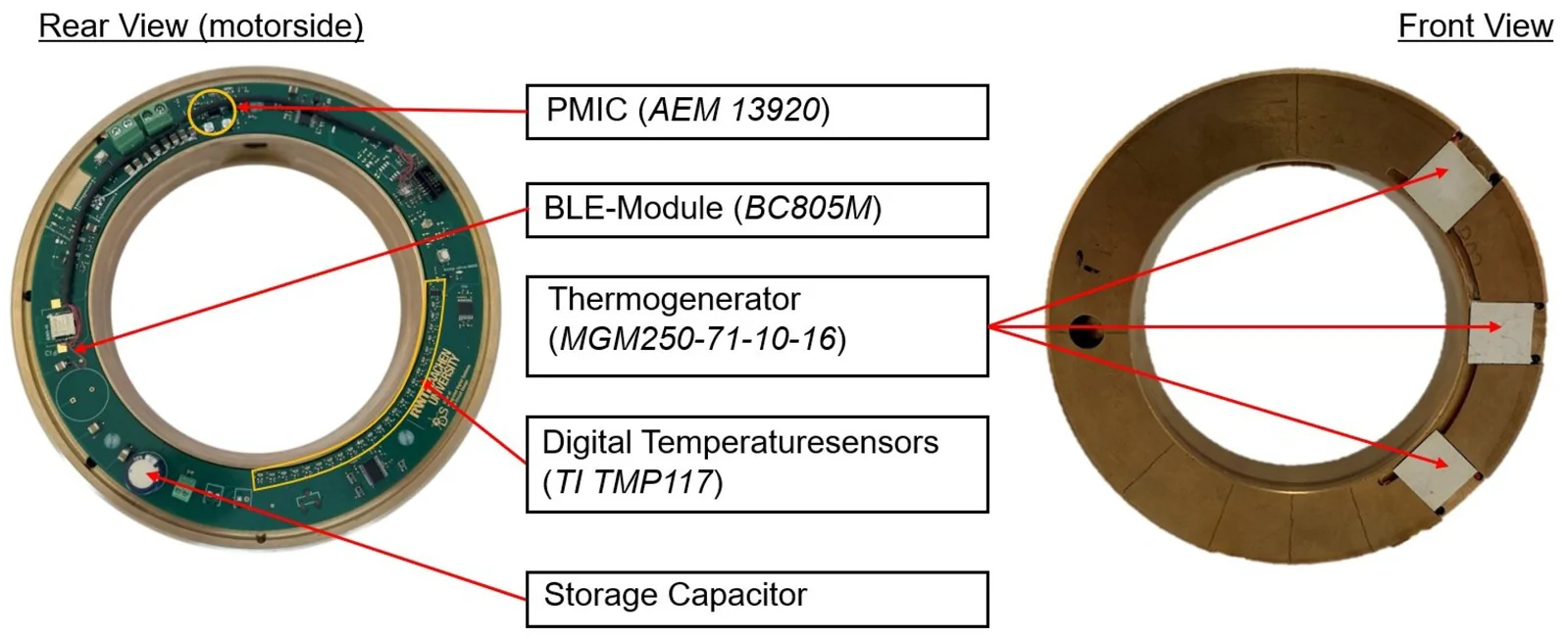
SiPB prototype (for detailed technical specifications of the device, cf. Table 1).

Sensor-integrating machine elements (SiME) are standard machine parts that have been instrumented with sensor electronics for in-place measurements [27]. Important features of SiMEs are energy autarky, wireless data transmission, the preservation of their primary function, and neutrality towards the installation space. This paper focuses on a sensor-integrating plain bearing (SiPB) that integrates temperature sensors along its inner perimeter to detect operational anomalies and predict failure. The prototype is illustrated in Figure 2. This prototype is an iteration of a previous design described in [28].

**Figure 3:**
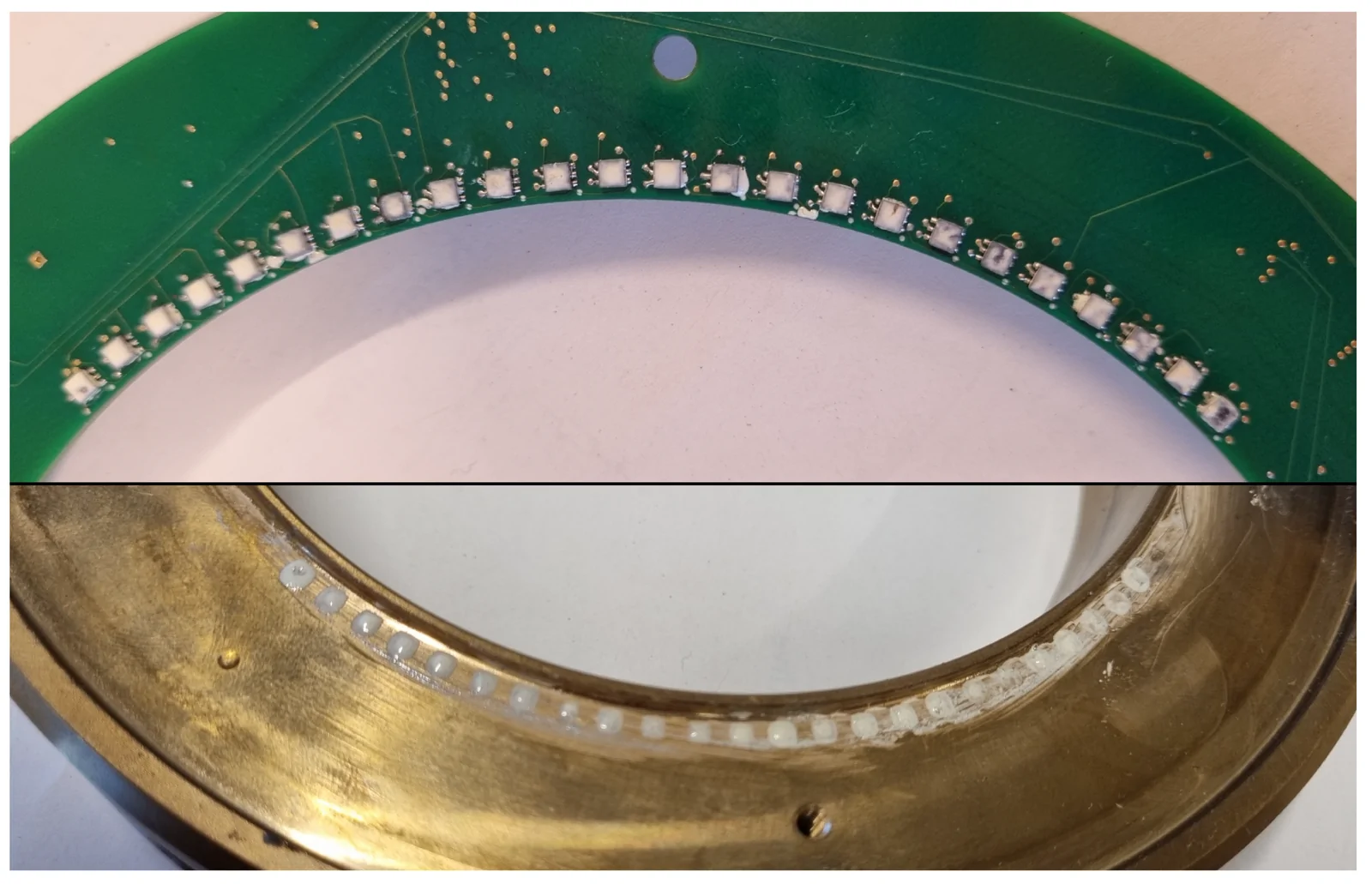
Thermal contact validation via contact pattern test.

The plain bearing is manufactured from CuSn12Ni2-C and features an inner diameter of $d_{i}=120\;\mathrm{mm}$, an outer diameter of $d_{a}=180\;\mathrm{mm}$, and a width of b=30mm. Before each test begins, a new hardened steel shaft sleeve is installed, which is in contact with the test bearing. The bearing clearance is e≈140μm. A recess with a depth of 2 mm was milled into the end face to integrate the PCB, including the temperature sensors and electronic components, into the bearing volume. The PCB is attached to the bearing with non-metallic screws. Thermal paste is applied beneath the temperature sensors to ensure proper thermal coupling to the bearing. The thermal contact was validated by a contact pattern test, as seen in Figure 3. On the back side, three additional recesses were milled to accommodate thermoelectric generators (TEGs). Matching the limited space, three 22 mm by 22 mm MGM250-71-10-16s (European Thermodynamics Limited, Leicestershire, UK) were used [29]. The sensor electronics comprise 24 TMP117 (Texas Instruments (TI), Dallas, TX, USA) [30] temperature sensors, an nRF52805 SoC (Nordic Semiconductor, Trondheim, Norway) [31] for data acquisition and transmission via Bluetooth Low Energy (BLE), and an AEM13920 PMIC (E-peas S.A., Louvain-la-Neuve, Belgium) [26] for thermal energy harvesting from the TEGs. A power management integrated circuit (PMIC) bundles multiple different functions required for power management on a single chip. The AEM13920 is specifically designed for energy harvesting. In Table 1 the key specifications of the integrated sensor system, power management components, and energy harvesters of the SiPB prototype are summarized.

To minimize static offsets between individual sensor channels, the relative calibration of the TMP117 array is performed before each measurement. At the initial time step (t=0), under steady-state conditions at room temperature, individual sensor offsets were adjusted so that all 24 sensors output their common arithmetic mean. While this single-point alignment eliminates initial offsets and significantly improves the relative channel-to-channel accuracy near ambient conditions, thermal drift and gain tolerances across the operating temperature range cannot be fully quantified without multi-point calibration. Therefore, the channel-to-channel accuracy must be assumed to be two times the absolute accuracy of ±0.2 °C [30].

#### 2.2.2. Test Rig Configuration

**Figure 4:**
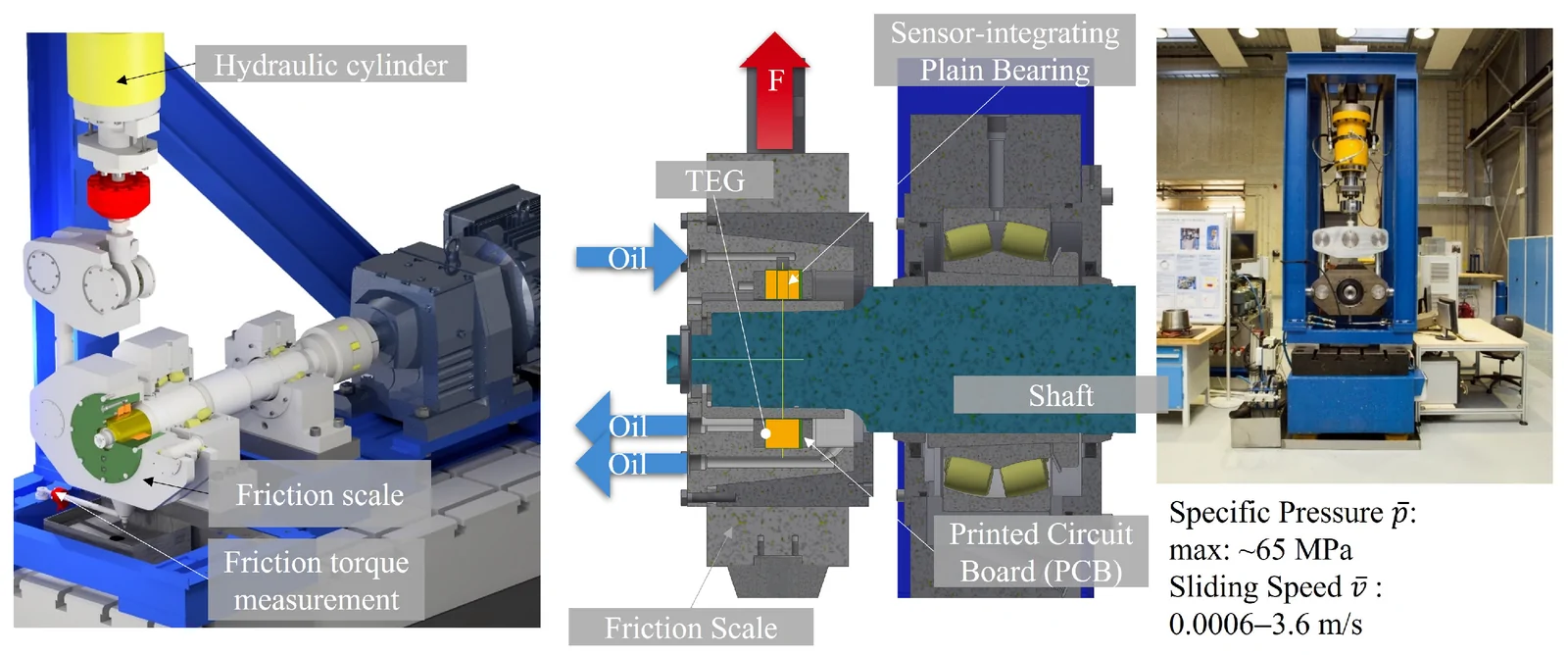
The 120 mm hydrodynamic radial plain bearing test rig (GLP120).

The experimental investigations conducted with the SiPB, the results of which are described in Section 3 below, were performed on a test rig for hydrodynamic radial plain bearings with an inner diameter of $d_{i}$ = 120 mm. Figure 4 displays a schematic diagram of the 120 mm plain bearing test rig (GLP120).

The SiPB is located at the front of the shaft and is radially loaded via a hydraulic cylinder. The hydraulic cylinder is capable of applying a maximum radial force of $F_{R}$ = 250 kN, which corresponds to a specific contact pressure of approximately ${\bar{p}}_{max}$≈ 65 MPa. The SiPB is located in a metal housing referred to as a friction balance. The friction balance is mounted on a pivot. Consequently, the friction torque $M_{R}$ prevailing in the SiPB is measurable using a lever arm and a connected load cell. The maximum friction torque measurable in the test bearing of the GLP120 is $M_{R,limit}$ = 550 Nm. For the experiments described in Section 3, a threshold value of $M_{R,max}$ = 450 Nm was selected as the criterion for the termination of the test. The shaft is supported by two roller bearings, whose housings are connected to the machine bed. The shaft is connected to a 250 kW electric motor via a jaw coupling. The electric motor enables sliding speeds of the shaft of up to $\bar{v}$ = 3.6 m/s. Heating elements are located in the friction balance, allowing the test bearing to be heated to defined temperatures. Additionally, the lubricating oil supply into the test bearing can be heated both in the tank and in the oil supply hose. The maximum temperature to which the oil in the tank and the friction balance can be heated is $T_{Initial}$ = $T_{oil,max}$ = $T_{fb,max}$ = 110 °C. A polyalphaolefin (PAO)-based industrial gear oil of ISO VG Class 320 (Castrol Optigear CT 320, Castrol, Rotterdam, The Netherlands, [32]) is used as the lubricating oil. This lubricating oil is characterized by a high load-carrying capacity and is used in heavy-duty drives, such as wind turbines.

**Figure 5:**
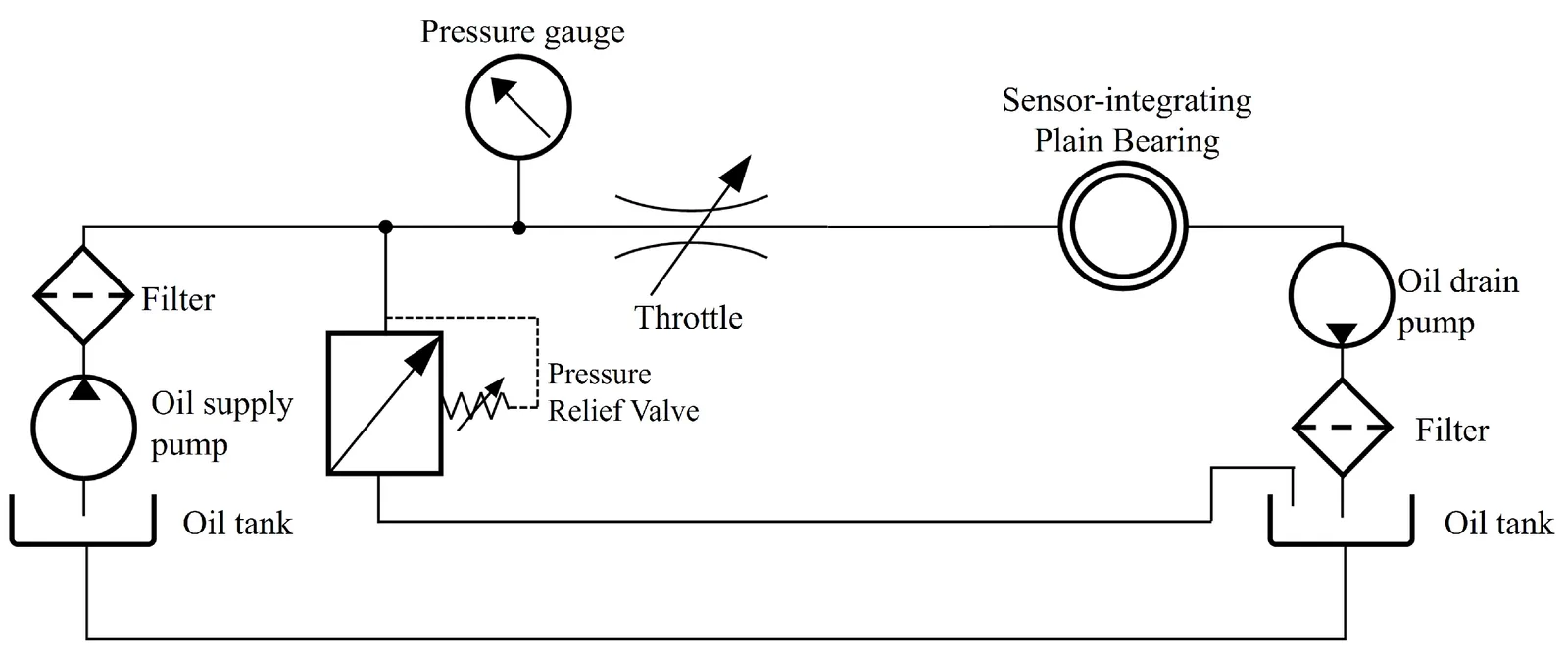
Schematic illustration of the regular oil circuit (GLP120).

Figure 5 schematically illustrates the regular oil circuit of the GLP120 test rig. The configuration shown is used for the experimental investigations of bearing failure due to overheating described in Section 3.1.1. Starting from the tank, the oil is delivered by the pump and maintained at a specified supply pressure via a pressure-limiting valve. The pressure gauge displays the current pressure before a downstream throttle directs the oil into the test bearing at a specified pressure ($p_{oil,in}$ = 0.2 MPa). The flow into the bearing is laminar, and the oil flow rate is approximately 8.5 L per minute.

The standard oil circuit of the test rig contains two particle filters, which continuously remove particles from the lubricant. Therefore, a separate contamination circuit was used for the particle contamination tests. It includes an intermediate tank with a stirrer to keep the particles suspended and a particle sensor in the oil supply line to monitor the particle concentration actually reaching the bearing.

**Figure 6:**
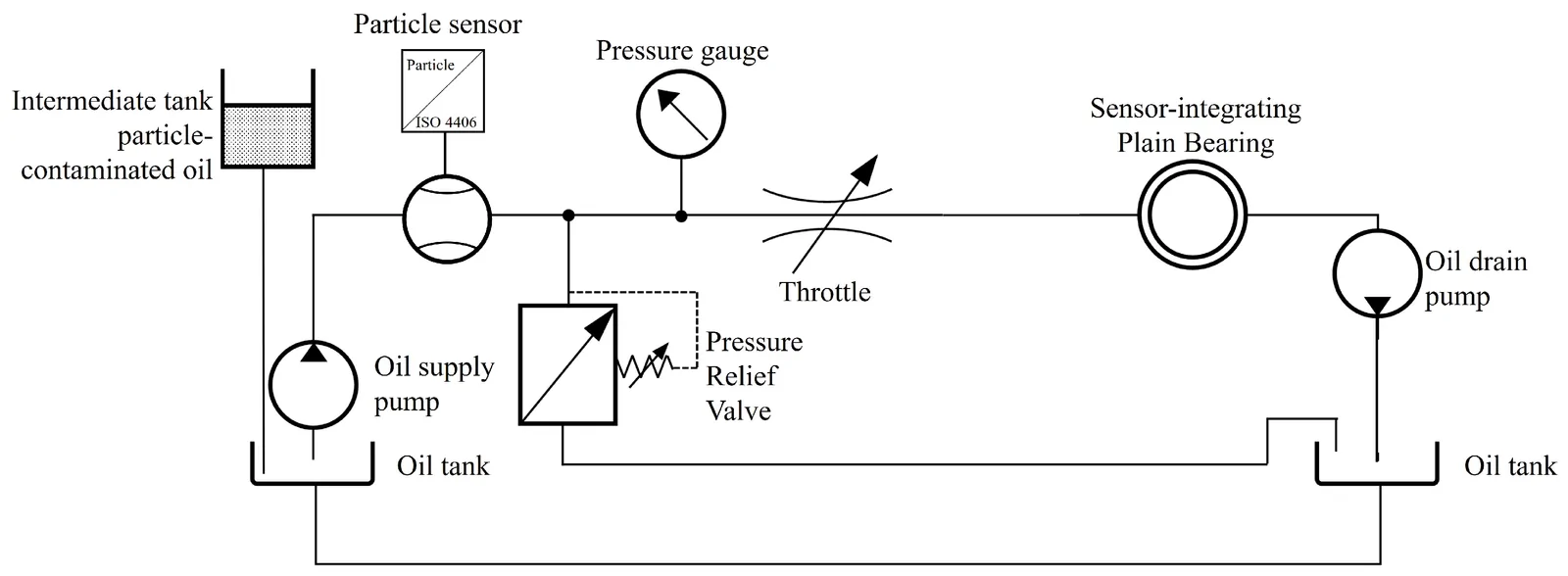
Schematic illustration of oil circuit for the contamination test (GLP120).

Figure 6 shows the modified oil circuit used for the particle contamination tests. This alternative setup was used for the experimental investigations described in Section 3.1.2.

Unlike the standard oil circuit configuration, no particle filters were installed in the oil circuit for the particle contamination tests. A stirring element was installed in the oil tank to prevent particles from settling at the bottom. The total oil volume in the test-bench setup was 25 L. To introduce particle contamination, 2 L was removed from the total volume and fed into the intermediate tank. The particles were then added to the oil in the intermediate tank. Iron dust was used as the particles added to the contaminated oil. The average particle diameter was in the range of 4–6 μm. A particle contamination level of $c_{particles}$ = 0.1 g/100 g, relative to the total oil volume, was introduced, analogously to [33]. The oil circuit passes through a particle sensor (CJC CMU [34]), which measures the number of particles, sorted by particle diameter per unit volume of oil passed through. Using a pressure gauge and a throttle valve, the oil is fed into the test reservoir at a defined pressure ($p_{oil,in}$ = 0.2 MPa). After the oil passes through the test reservoir, it is returned to the tank via a return pump. Table 2 summarizes the characteristics of the test-bench setup.

#### 2.2.3. Data Analysis

The temperature field is recorded by *n* = 24 temperature sensors distributed over the angular sector φ = 180°–266.25° (pitch 3.75°). In all TFM tests (overheating and particle contamination), the temperature field was sampled at a sampling rate of $f_{s}$ = 2.5 Hz.

The following parameters are derived from the temperature field. The global temperature gradient is defined as the difference between the maximum and the minimum temperature of all sensors at time *t*: $\Delta T_{global,t}=T_{max,t}-T_{min,t}$. The angular position of the maximum temperature $\varphi_{T,max}$ is the angular position of the sensor showing $T_{max,t}$. The local temperature change $\Delta T_{local,\varphi ,\Delta t}$ is the temperature difference at a fixed angular position φ over the time interval Δt: $\Delta T_{local,\varphi ,\Delta t}=T_{\varphi }\left(t\right)-T_{\varphi }\left(t-\Delta t\right)$. For the particle contamination test, the mean temperature of all sensors is calculated at each time step. The interquartile range (IQR) is evaluated over a window of $t_{w}$ = 60 min (corresponding to 9000 samples at $f_{s}$ = 2.5 Hz) as described in Section 3.1.2. The IQR is defined as $IQR=Q_{3}-Q_{1}$, where $Q_{1}$ and $Q_{3}$ are the 25th and 75th percentiles of the sorted samples within the window [35].

The characteristic parameters were determined in the post-processing of the recorded temperature field data. The IQR was defined after the tests had been conducted and was calculated from the stored time series of the mean temperature for the time segments shown in Figure 15 ($t_{w}$ = 60 min, i.e., 9000 samples at $f_{s}$ = 2.5 Hz) as $IQR=Q_{3}-Q_{1}$. The IQR was therefore not computed on the microcontroller during the tests.

Since the IQR requires only the quartiles of a sample, it can also be determined on the bearing-integrated microcontroller (nRF52805 SoC, 24 kB RAM). Storing and sorting 9000 values is not required: the mean temperatures of a window are counted in a histogram with a bin width of 0.01 °C, which is small compared to the observed IQR values (0.07–0.18 °C). At the end of each window, $Q_{1}$ and $Q_{3}$ are read from the cumulative bin counts. This requires only integer arithmetic and approximately 1 kB of RAM for 500 bins of 16 bits and negligible computational time. The on-board computation was not implemented in the present study; its implementation and validation against the post-processed IQR will be part of future work.

## 3. Results

This section presents the experimental validation of the developed SiPB prototype. First, in Section 3.1, the developed temperature field-based condition monitoring approach is validated via the GLP120 test rig for the two abnormal events: overheating and particle contamination. Section 3.2 examines the energy budget of the SiPB prototype in a laboratory setup. First, the energy demand is presented as a function of the measurement strategy. Based on this knowledge of the energy demand, the section closes with an investigation of the temperature differences that must exist at the TEG in order for the necessary energy to be obtained via thermoelectric energy harvesting.

### 3.1. Temperature Field Measurement

#### 3.1.1. Overheating Due to Mechanical Overload

Tests were conducted with a high energy input into the SiPB, far exceeding regular operating conditions. This was done despite the use of a highly additive-enhanced lubricating oil for heavy-duty drives and a regular lubrication supply. The goal is to induce mechanical overload and provoke a thermal imbalance in the test bearing. This imbalance ultimately leads to rapid bearing failure due to overloading. Such conditions allow the sensitivity and response speed of the CMS to be evaluated. Unlike the contamination test described in Section 3.1.2, the overloading tests do not involve a preliminary run-in procedure to smooth out roughness peaks. No run-in procedure was used in this part because, with run-in test bearings, it was not possible to induce overload damage under the operating conditions that were feasible on the test rig.

**Figure 7:**
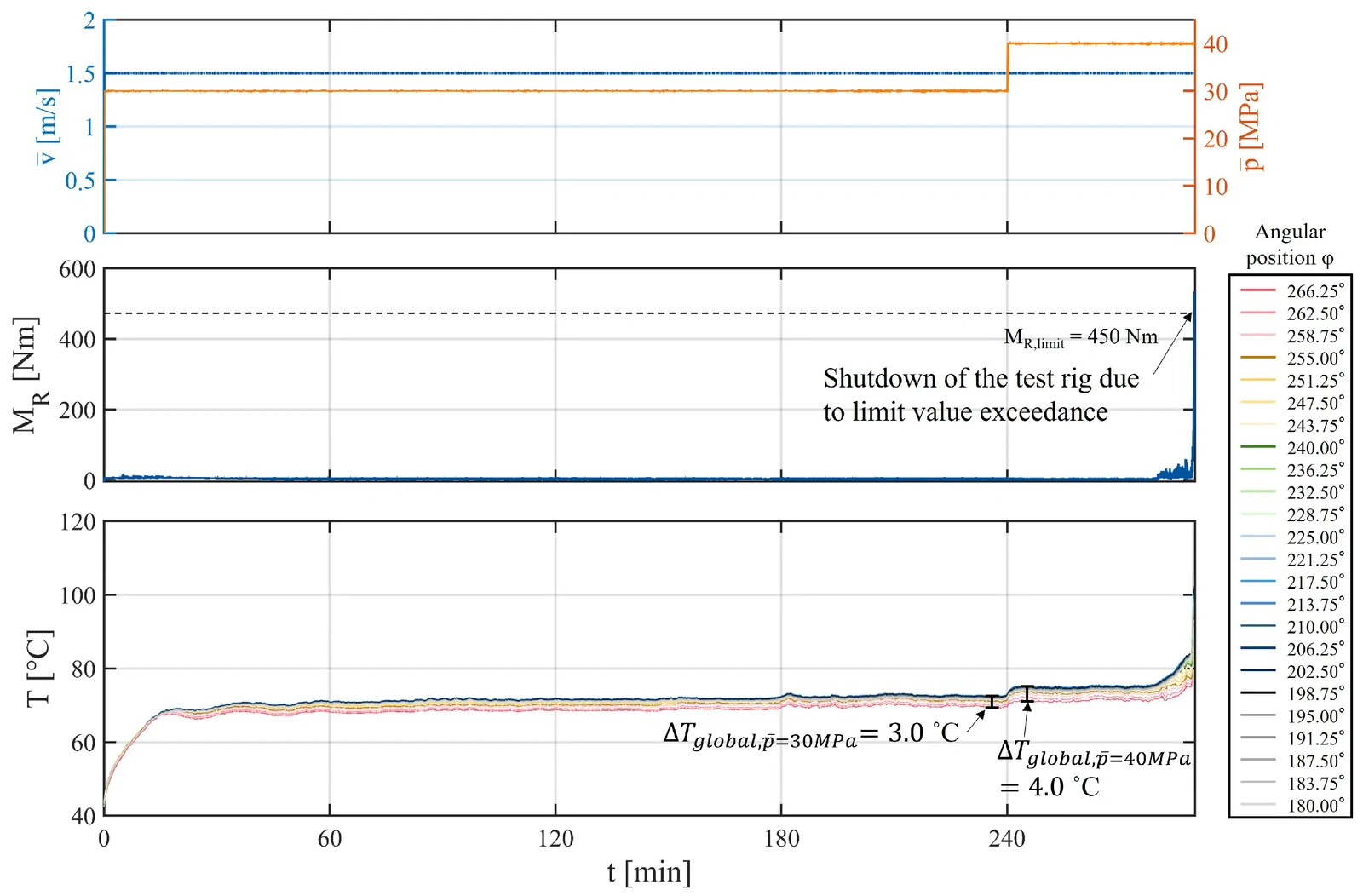
Experimental procedure for inducing overheating damage through mechanical overload ($\bar{p}$ = 30; 40 MPa, $\bar{v}$ = 1.5 m/s, $T_{Initial}$ = 60 °C).

Overloading and the resulting overheating of the bearing are subsequently caused by a high specific pressure $\bar{p}$ and high sliding speed $\bar{v}$ over an extended period of time. Figure 7 displays the $\bar{v}$-$\bar{p}$ curves (top), the friction torque $M_{R}$ curve (middle), and the TFM curve (bottom) over the testing time.

The temperature of the lubricating oil and the friction balance was controlled to $T_{Initial}$ = 60 °C. The sliding speed remained constant at $\bar{v}$ = 1.5 m/s throughout the entire test. The initial specific pressure was $\bar{p}$ = 30 MPa and was maintained for *t* = 240 min. After a test duration of *t* = 240 min, the specific pressure was increased to $\bar{p}$ = 40 MPa and subsequently maintained at a constant level. After a test duration of *t* = 289.7 min, the test was terminated due to the friction torque limit value $M_{R,limit}$ = 450 Nm being exceeded.

**Figure 8:**
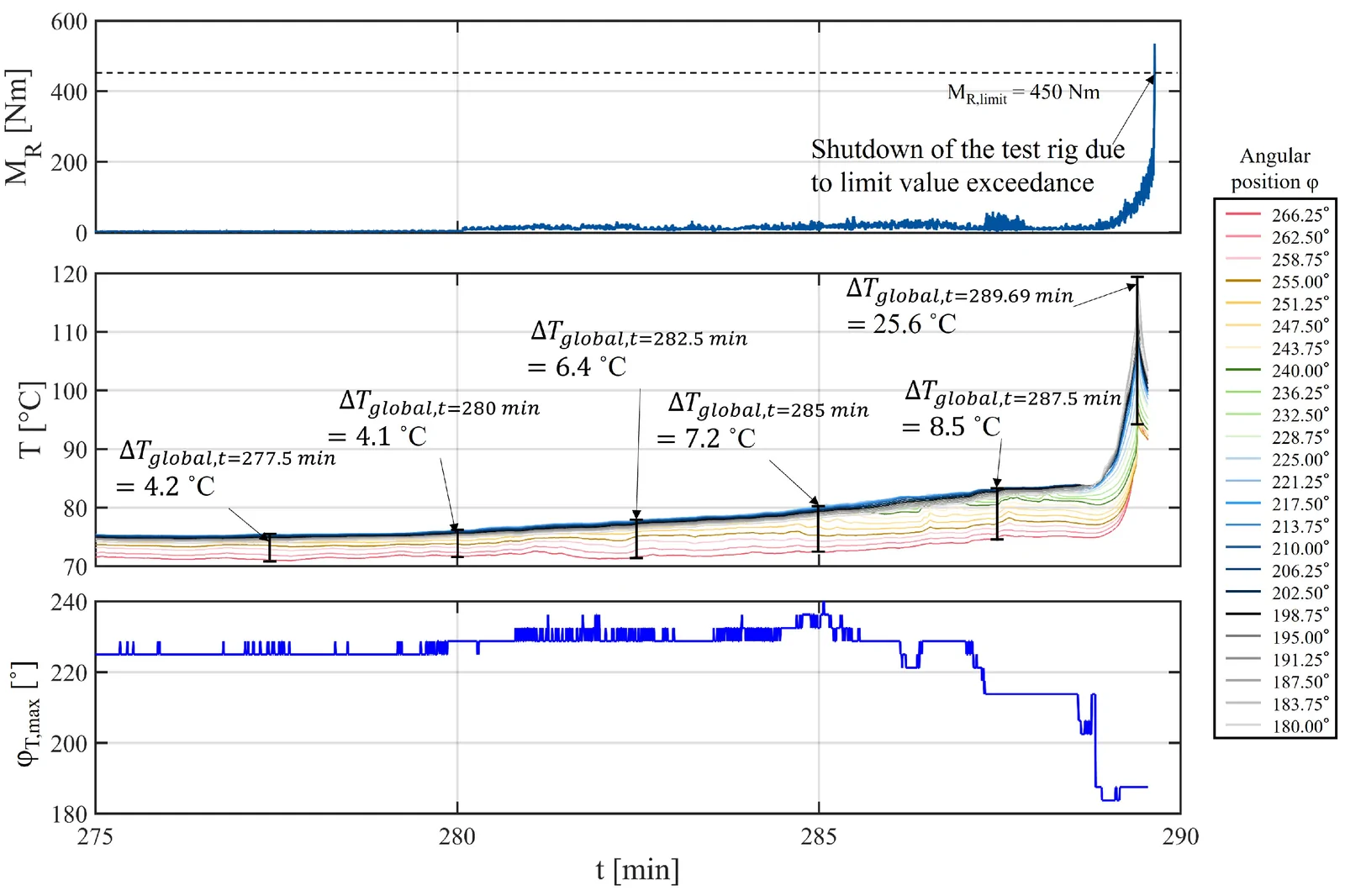
Friction torque, TFM, and $\varphi_{T,max}$ curves (*t* = 275–290 min) corresponding to Figure 7.

Following an initial rise in temperature caused by the startup process, a nearly steady-state thermal condition develops in the temperature field at a specific pressure of $\bar{p}$ = 30 MPa after approximately *t* = 20 min of testing. Suitable TFM-based parameters for assessing damage criticality are $\Delta T_{global,t}$ and $\Delta T_{local,\varphi ,\Delta t}$. The temperature level across all measurement positions and the global temperature gradient $\Delta T_{global}$ remains stable until the load is increased. As the load increases (*t* = 240 min), a moderate temperature rise $\Delta T_{local,\Delta t=4min}$ = 1.8–2.9 °C occurs across all angular positions. The global temperature gradient increases with the load increase from $\Delta T_{global,\bar{p}=30MPa}$ = 3.0 °C (*t* = 235 min) to $\Delta T_{global,\bar{p}=40MPa}$ = 4.0 °C (*t* = 245 min). Starting at *t* = 280 min into the test, increases in the friction torque of up to $M_{R}$ = 50 Nm ($\mu =1.19\times 10^{-3}$) are evident. Concurrently, localized temperature increases can be observed. For better visibility, Figure 8 displays the friction torque (top), temperature field (middle), and $\varphi_{T,max}$ (bottom) curves for the time range *t* = 275–290 min.

Local temperature $\Delta T_{local}$ increases can be observed with the earliest increases in friction torque. In particular, these temperature increases are evident at the measurement positions where high baseline temperatures were already present before the increase in friction torque. In the time interval *t* = 280–285 min, the minimum temperature $T_{min}$ remains nearly constant, while the maximum temperature $T_{max}$ rises linearly over time. The occurring increases in friction torque can be interpreted within the range of up to *t* = 289 min as recurring, local contacts between roughness peaks on the bearing and the shaft. Due to the high thermal load, the separating lubricating film breaks down locally, causing mixed friction to occur. The TFM is sensitive enough to detect these occurring friction phenomena with a local resolution. This hypothesis is also supported by the curve of the angular position of the maximum temperature $\varphi_{T,max}$ shown in Figure 8. Starting at time *t* = 280 min, a discontinuity in the $\varphi_{T,max}$ curve can also be observed with the onset of mixed friction. Starting from the previously fairly constant level of $\varphi_{T,max}$ = 225° (*t* < 280 min), the maximum temperature subsequently shifts to a higher angular level (approximately $\varphi_{T,max}$ = 232.5°). From *t* = 286 min, $\varphi_{T,max}$ drops to a lower level and initially remains at $\varphi_{T,max}$ = 213.75°. From *t* = 289 min, the friction torque rises rapidly and exceeds the set limit value $M_{R,limit}$ = 450 Nm at time *t* = 289.69 min. From *t* = 289 min onwards, the temperatures rise sharply at all angular positions. The global temperature gradient at the shutdown time is $\Delta T_{global,t=289.7min}$ = 25.6 °C. Additionally, $\varphi_{T,max}$ drops rapidly to a value of $\varphi_{T,max}$ = 187.5° due to the intense mixed friction in the load zone. Table 3 displays the characteristic values discussed for the time range *t* = 277.5–289.7 min. Operating points with increased friction torque values are highlighted in grey in Table 3. In addition, Figure 8 shows discontinuities in the temperature signal, which shift over time from high φ-values to low φ-values. This phenomenon is discussed in detail at the end of this section (Section 3.1.1).

**Figure 9:**
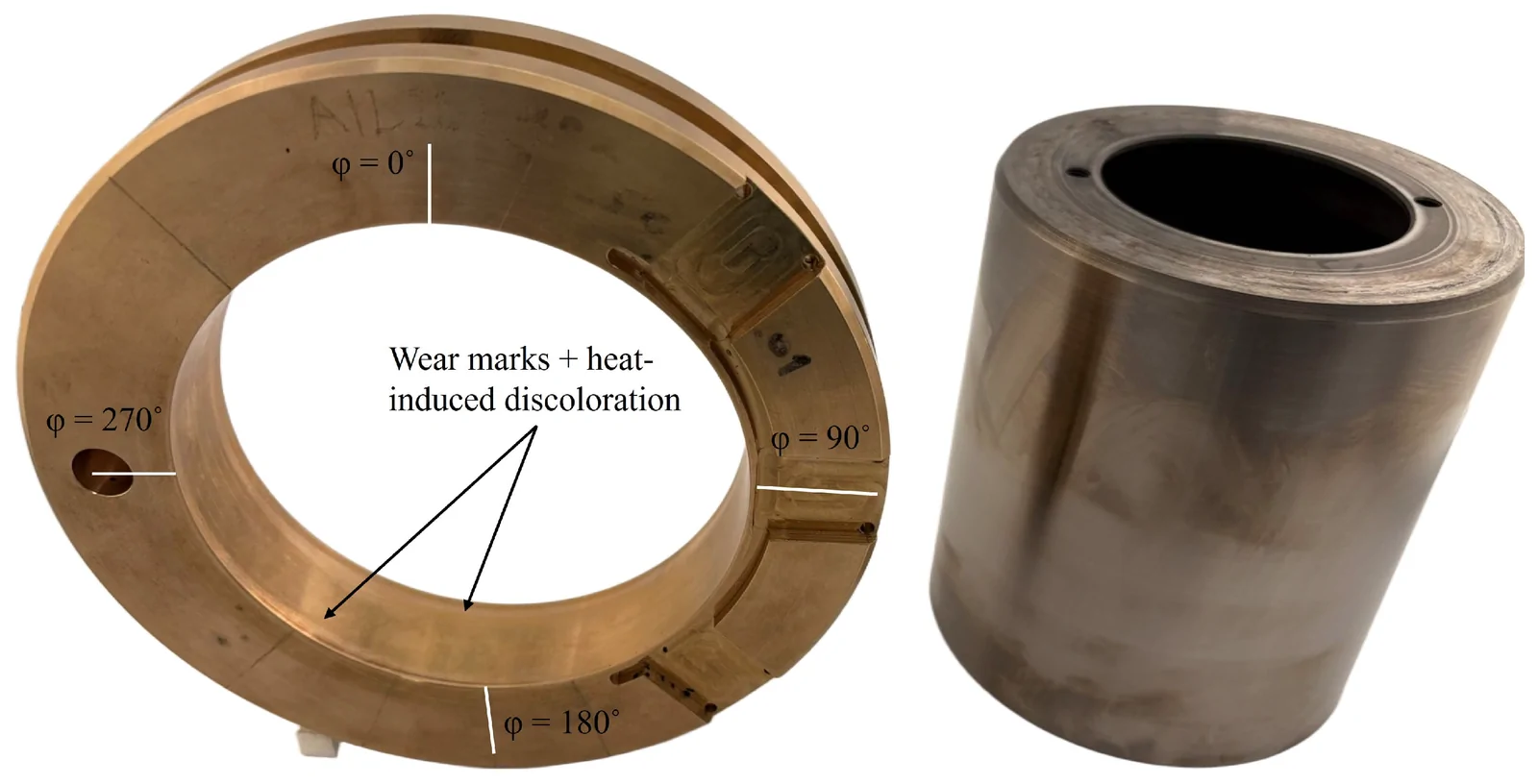
Overheating of bearing after test in Figure 7.

Figure 9 depicts the test bearing and shaft sleeve used in the experiment described in Figure 7 and Figure 8. Circumferential wear marks are visible on the bearing’s running surface near both end faces. In addition, heat-induced discoloration is visible on the end face, on the running surface, and directly below the end face, which provide evidence of significant heat-related damage to the bearing.

**Figure 10:**
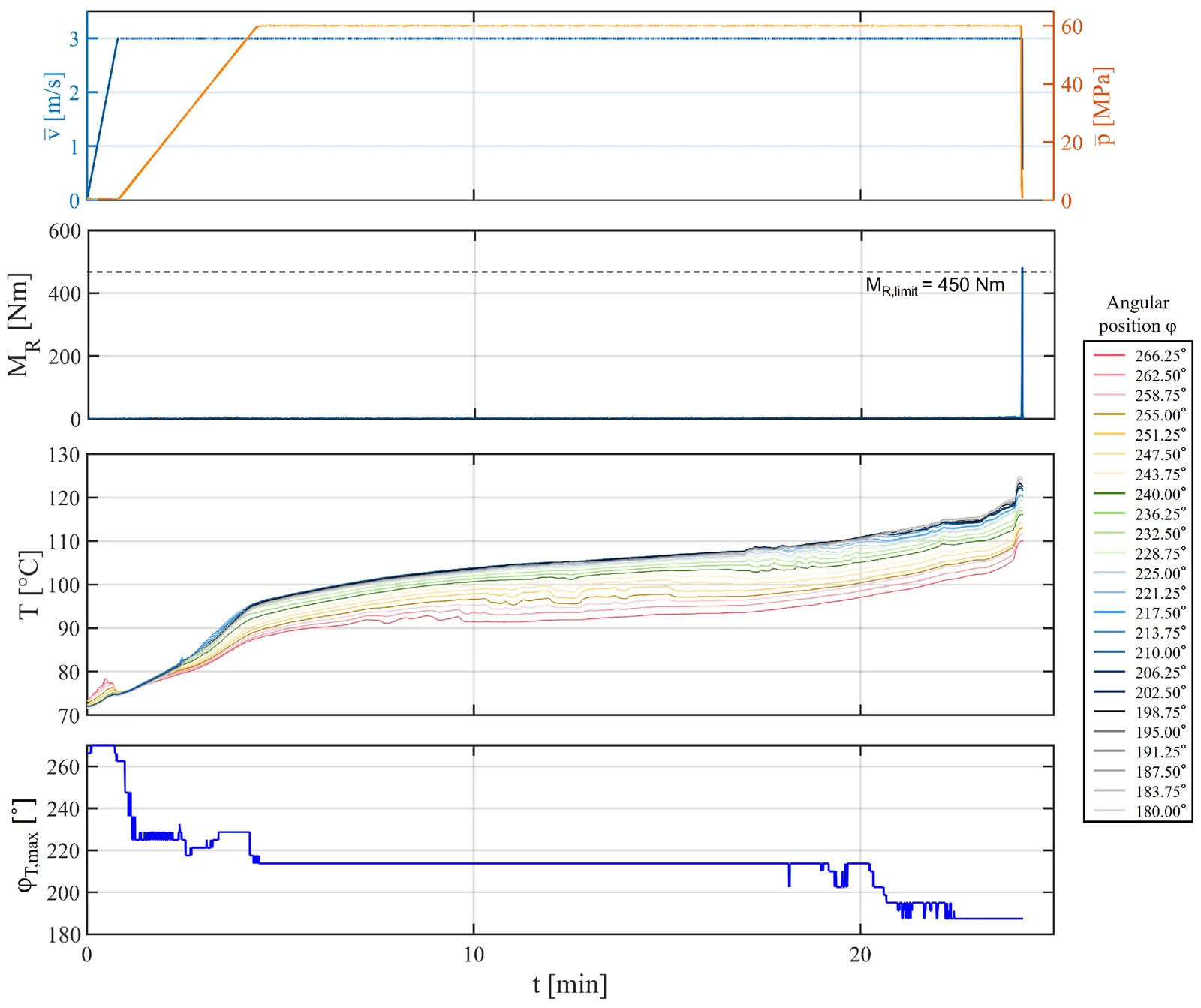
Overheating test 2 ($\bar{p}$ = 60 MPa, $\bar{v}$ = 3.0 m/s, $T_{Initial}$ = 80 °C).

Finally, this experiment indicates that even rapidly progressing overheating damage may be detected early using TFM. For this purpose, the test conditions were selected such that the thermal energy input into the test bearing was maximized to induce rapid overheating. The initial temperature was increased to $T_{Initial}$ = 80 °C. The sliding speed was $\bar{v}$ = 3 m/s and the specific pressure $\bar{p}$ = 60 MPa. Figure 10 displays the corresponding $\bar{v}$-$\bar{p}-$, friction torque, TFM, and $\varphi_{T,max}-$ curves from the test. The failure criterion for the test was selected to be the limit friction torque $M_{R,limit}$ = 450 Nm, analogously to the previous test.

Once the final load ($\bar{p}$ = 60 MPa; *t* = 4.4 min) is applied, a high $\Delta T_{global}$ value (>10 °C) develops quickly, indicating operation at a critical failure level. Throughout the entire test duration, $\Delta T_{global}$ remains at a constant level, as shown in Table 4. A comparison with Table 4 also indicates that the $\Delta T_{global}$-values in Table 4 are already at least twice as high in the phase without increased friction torques (white background). The increased $\Delta T_{global}$-values underline the critical nature of the second overheating test’s operating conditions, even before any increase in friction torque was measured. Times at which an increased friction torque occurs are highlighted in gray in Table 4.

**Figure 11:**
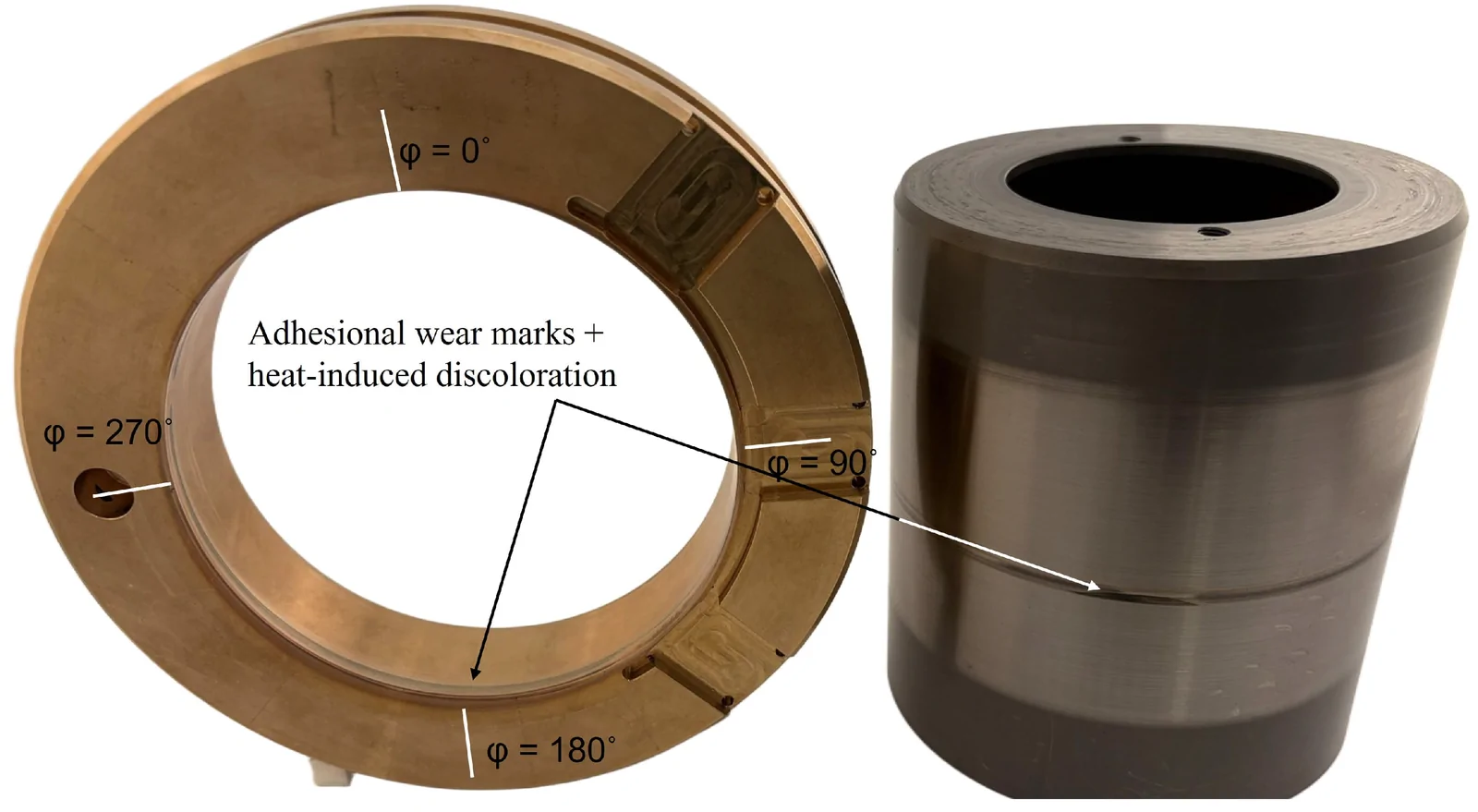
Test bearing and shaft sleeve after the test in Figure 10.

Throughout the entire test duration, the temperature rises continuously across all measurement positions, which also indicates a failure-critical condition of the test bearing. $T_{max}$ is therefore also a valid limit value for defining alarm thresholds based on TFM. Starting at *t* = 20 min, the $\varphi_{T,max}$ value shifts toward lower angular positions. At time *t* = 24.12 min, the friction torque spikes and exceeds the limit value $M_{R,limit}$ = 450 Nm. Prior to this, no increased friction torque values compared to the typical friction torque values in the fluid friction range were present. Figure 11 depicts the test bearing and shaft sleeve used in the experiment described in Figure 10.

In both overheating tests, $\varphi_{T,max}$ shifts to 187.5° shortly before the friction torque limit is exceeded (Figure 8 and Figure 15). This is only 7.5° from the edge of the sensor array (180°). It cannot be excluded that the actual temperature maximum was located outside the instrumented sector in this phase. The $T_{max}$ and $\Delta T_{global}$ measured by the SiPB could therefore represent lower bounds on the actual values considering the temperature field across the entire bearing angle.

**Figure 12:**
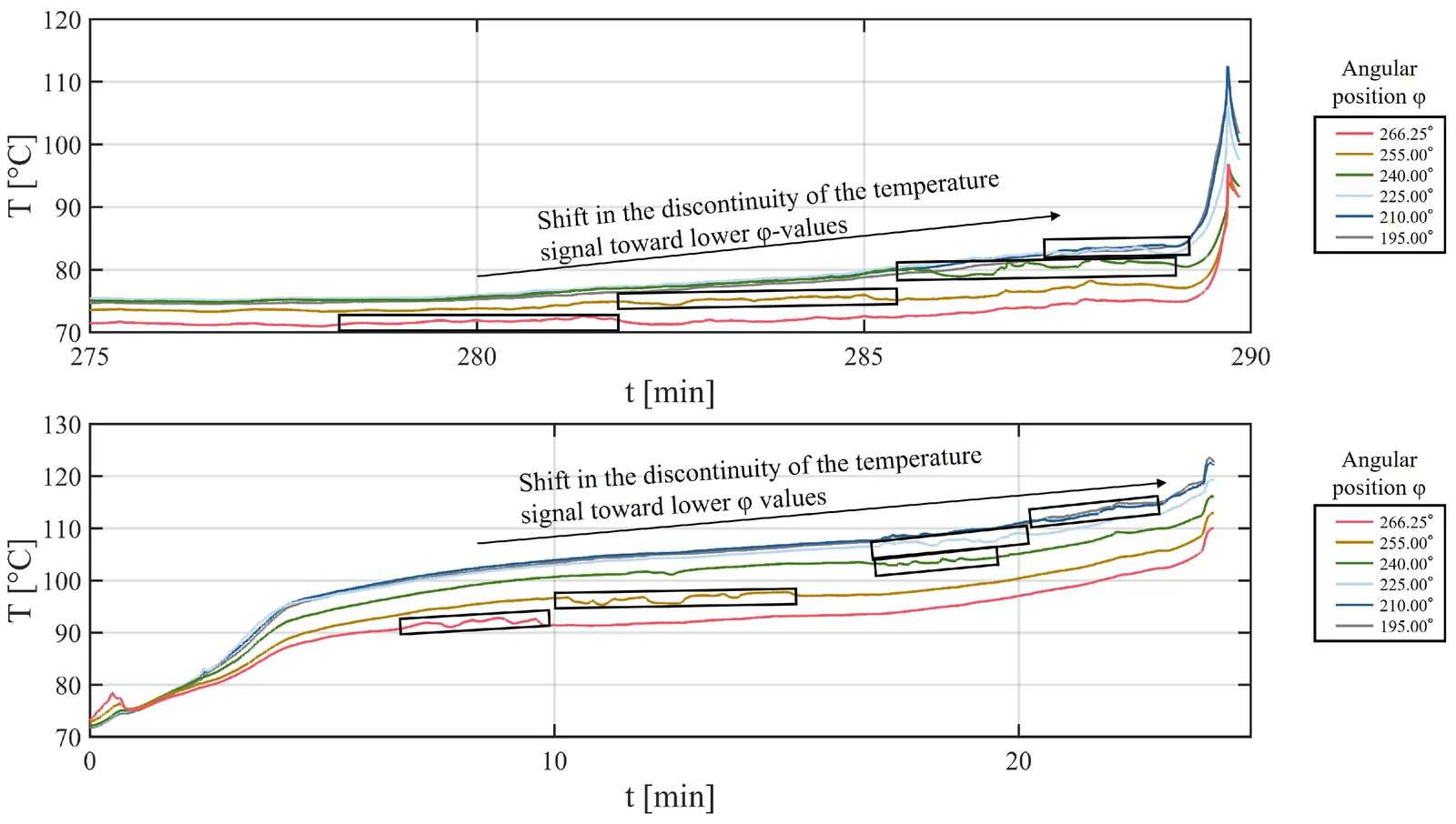
Overheating unsteadiness within the temperature field curves.

For the two overheating tests described, a oscillating signal discontinuity pattern can be observed in the temperature field measurement prior to the onset of damage. The signal discontinuity occurs over a period of approximately Δt = 3–6 min at a specific angular position φ and may potentially indicate an unstable thermal operating condition. Figure 12 depicts the discontinuities in the temperature signals for the two tests. For better visibility, only six measurement positions of the temperature field (φ = 195°; 210°; 225°; 240°; 255°; 266.25°) are plotted.

The signal discontinuities are outlined in black in Figure 12. It can be seen that, over time, the discontinuities initially occur at high φ values and low temperature levels. Over the course of the experiment, the signal discontinuity shifts to lower φ values and higher temperature levels. The described discontinuities in the temperature field occurred only in the two experiments described and thus represent another potential characteristic for early damage detection in cases of overheating damage caused by mechanical overload. In both of the overheating tests carried out, the signal discontinuities occur at least 10 min before the friction torque limit $M_{R,limit}$ is exceeded. However, further experimental investigations are necessary to clearly determine the underlying physical effects that cause these temperature signal discontinuities.

#### 3.1.2. Particle Contamination

**Figure 13:**
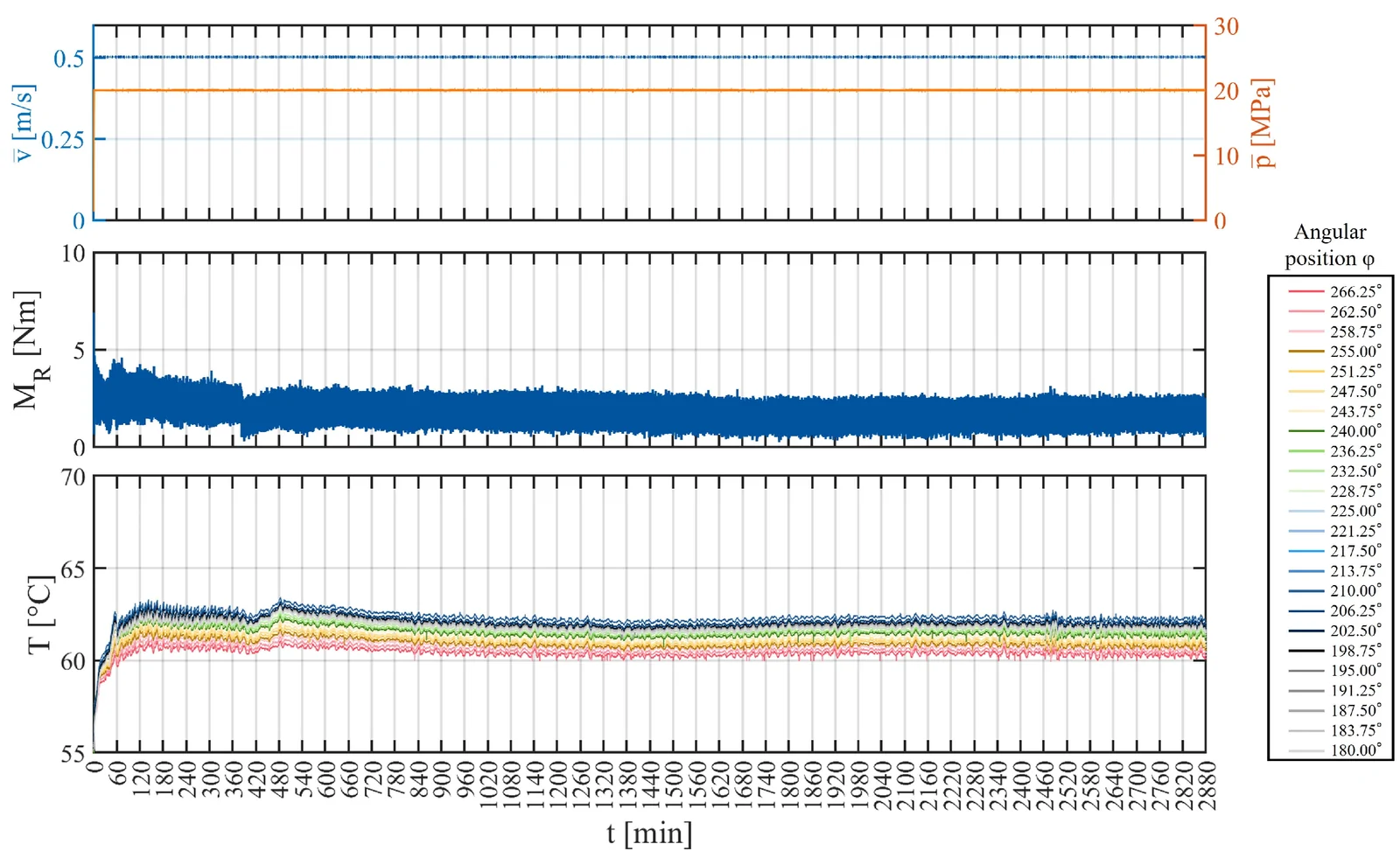
Running-in procedure: particle contamination test ($\bar{p}$ = 20 MPa, $\bar{v}$ = 0.5 m/s, $T_{Initial}$ = 60 °C).

This subsection evaluates the suitability of the TFM approach for detecting particle contamination in the lubricant. For the experiment, a constant operating point is first maintained for *t* = 2880 min (48 h) as a preliminary running-in test. The aim of the running-in test is to achieve an operating point in the fluid friction region at the time of particle contamination, while maintaining the lowest possible lubrication gap height. If the minimum lubrication gap height $h_{min}$ is too high during particle contamination, it is expected that the particles will not be able to pass through the lubrication gap unimpeded without coming into contact with the surfaces. This test procedure ensures that any potential running-in processes of the test bearing are completed. The specific contact pressure was $\bar{p}$ = 20 MPa and the sliding speed $\bar{v}$ = 0.5 m/s. The temperature of the friction balance and the oil tank was controlled to $T_{Initial}$ = 60 °C. Figure 13 depicts the friction torque curve (center) and the temperature field curve (bottom) associated with the running-in procedure.

Figure 13 shows that there is a slightly elevated friction torque ($M_{R}$ = 7 Nm) at the start of the test. The temperature rises moderately across all positions within the first 120 min. At the same time, the friction torque level decreases. After approximately *t* = 180 min, the temperature field has reached a constant distribution and remains nearly constant throughout the entire duration of the running-in test. The friction torque $M_{R}$ drops to a constant final value after approximately *t* = 480 min. Since both the temperature distribution and the friction torque reach constant levels, it can be concluded that the running-in process is complete before the end of the test.

**Figure 14:**
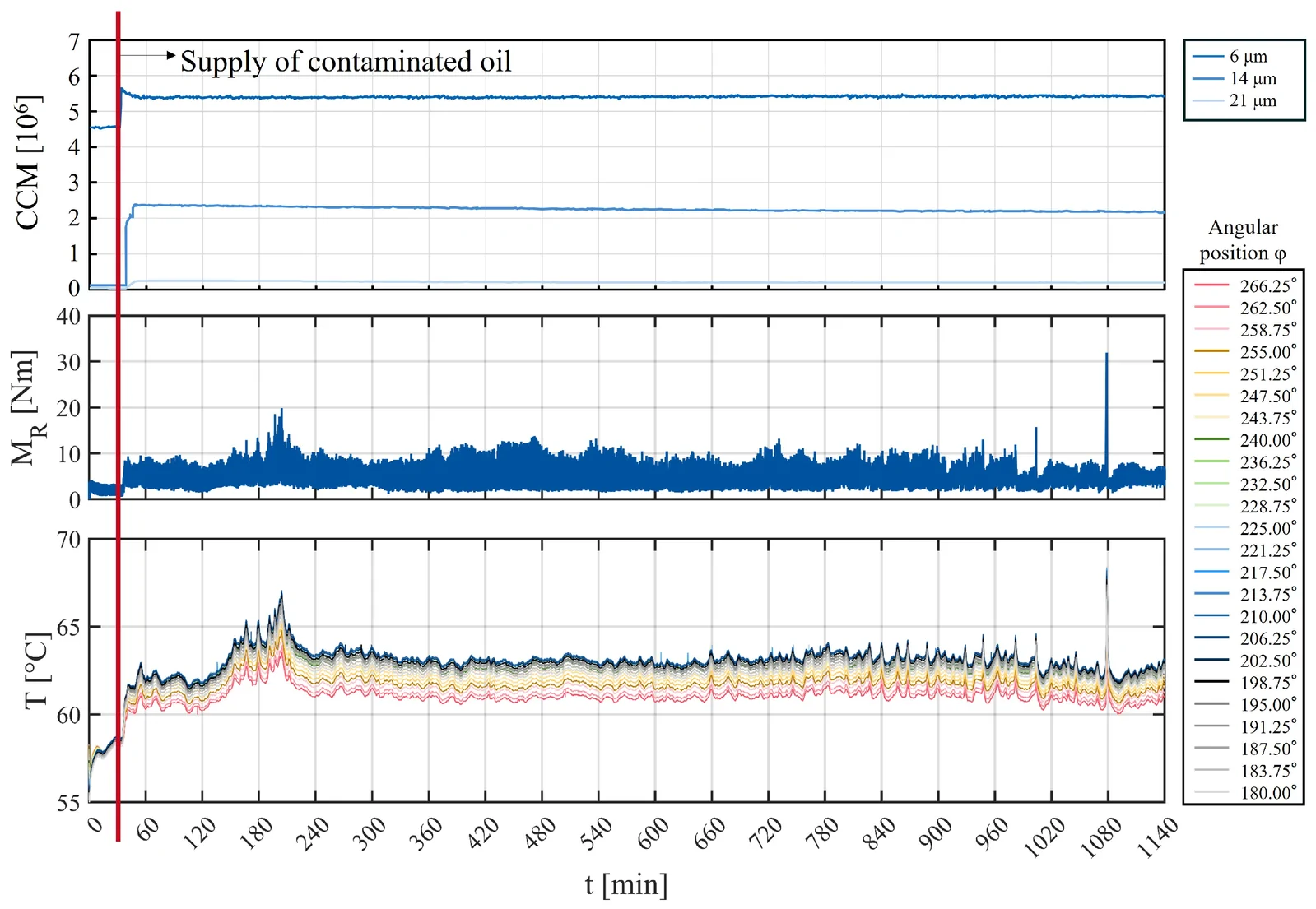
Particle concentration, friction torque, and temperature field over time ($\bar{p}$ = 20 MPa, $\bar{v}$ = 0.5 m/s, $T_{Initial}$ = 60 °C).

The test was then paused, and the contaminated oil was subsequently fed into the oil circuit setup shown in Figure 6. The contaminated oil was fed from an intermediate tank into the main tank at a specified time (see Figure 6). The test was then continued for another 24 h while measuring the friction torque, the particle count in the oil supply, and the temperature field. The total oil volume in the test bench setup was 25 L. To add the particle contamination, 2 L was removed from the total volume. Iron dust was used as the particles to contaminate the oil. The average particle diameter was in the range $d_{Particle}$ = 4–6 µm. Particle contamination of $c_{Particle}$ = 0.1 g/100 g relative to the total oil volume of 25 L was introduced. The specific pressure $\bar{p}$, the sliding velocity $\bar{v}$, and the initial temperature $T_{Initial}$ were selected analogously to the running-in test. Figure 14 displays the particle concentration detected by the particle counter in the oil supply (top), the friction torque curve (middle), and the temperature field (bottom) over time.

Immediately after the contaminated oil is introduced into the main tank of the oil circuit, an increase in particle concentration is observed at the particle counter, which is integrated into the oil supply line. CCM represents the absolute number of particles per liter of oil. Throughout the entire test duration, the particle concentration remains at a higher level than before contamination, although a slight decrease in particle concentration is observable over time. This slight decrease in the number of particles can be attributed to particle deposits in the hoses and in the oil tank. The friction torque rises quickly after particle contamination, increasing from a baseline level of $M_{R}$ = 2 Nm to approximately 6 Nm. This corresponds to an increase in the coefficient of friction of Δμ = $1.905\times 10^{-4}$, which corresponds to very light mixed friction. Over the entire subsequent time course, the friction torque signal remains discontinuous and exhibits local maxima of up to $M_{R}$ = 32 Nm (*t* = 1080 min). The temperatures rise by approximately $\Delta T_{local}$ = 4 °C across all measurement positions following particle contamination. The qualitative trend of the TFM corresponds very well with the friction torque curve. It can be seen that increased mixed friction caused by the prevailing three-body abrasion is accompanied by local temperature increases. The temperature level is slightly higher compared to the running-in test.

**Figure 15:**
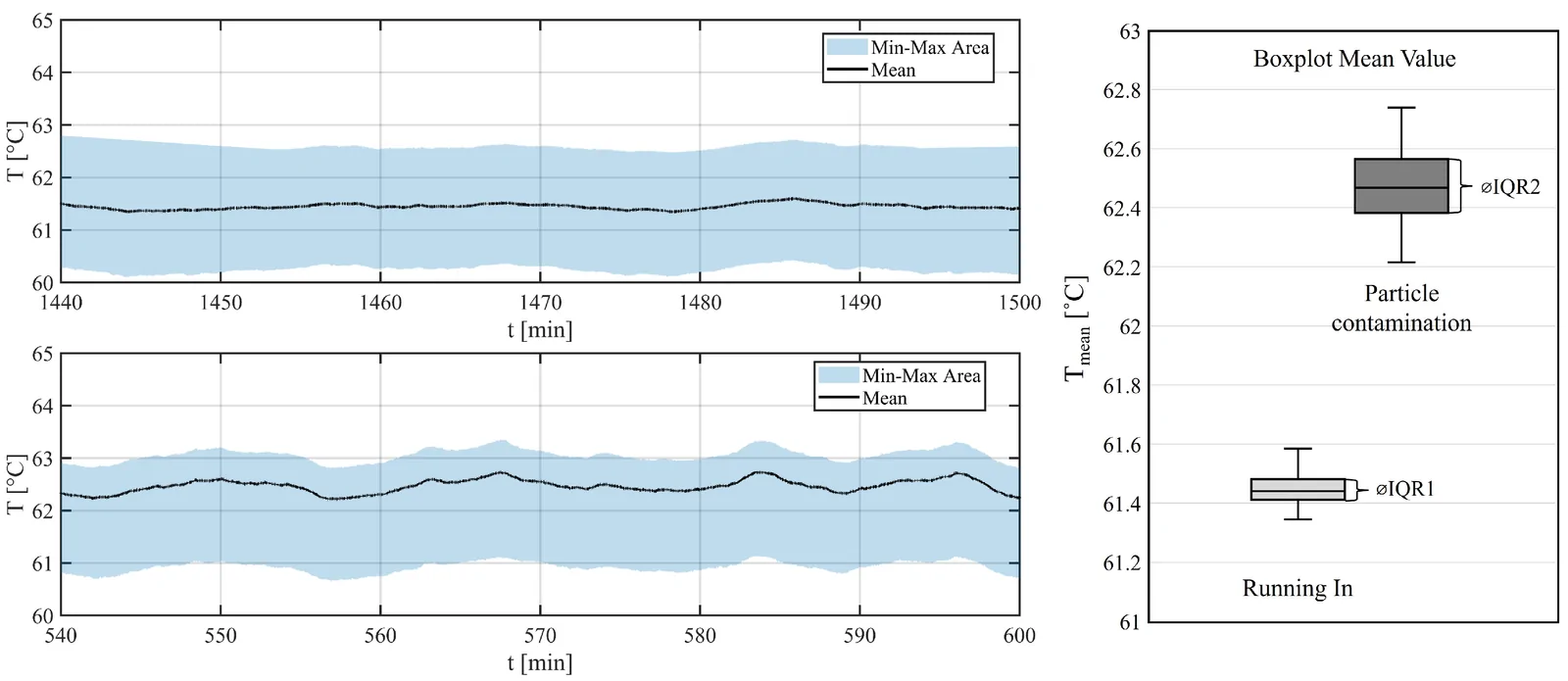
Comparison of TFM during running-in and contamination tests.

The temperature field profile also shows that the temperature curves are significantly more erratic than in the running-in test. Figure 15 displays a time segment of the temperature field for the running-in test (top) and the particle contamination test (bottom), respectively. For better visibility, only the mean value of the 24 temperature sensors (black) and the minimum and maximum temperatures of the temperature field (light blue) are shown. The right-hand side displays the boxplot distribution of the mean values for the time periods shown in Figure 15.

The interquartile range (IQR) of the mean temperature is a suitable measure for detecting discontinuities in the temperature field signal. The IQR is an established measure of dispersion in descriptive statistics [35]. To calculate the IQR, a sample is sorted by size. The IQR indicates the width of the interval in which the middle 50 % of the sample elements are located. For the test sections shown in Figure 15, the IQR of the mean temperature was determined. The IQR of the mean temperature for the particle contamination test shown in Figure 15 is ø${IQR}_{2}$ = 0.18 °C and thus is approximately 2.6 times higher than in the previous running-in test (ø${IQR}_{1}$ = 0.07 °C). The IQR of the temperature field mean therefore represents another suitable characteristic for the TFM-based detection of particle contamination. The bearing-integrated microcontroller enables the IQR of the temperature field mean to be continuously determined during operation.

**Figure 16:**
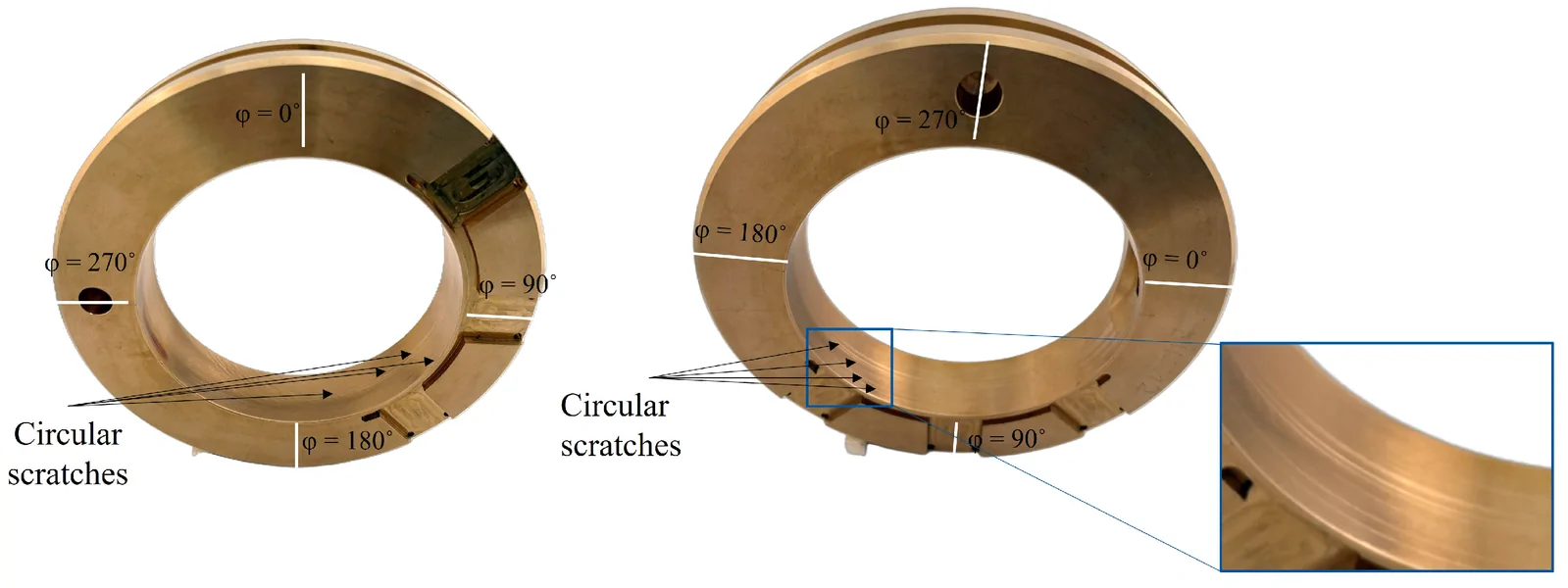
Test bearing after contamination test.

Figure 16 depicts the test bearing after the test was completed. It can be seen here that particle contamination caused three-body abrasion and, as a result, characteristic circumferential grooves [36].

### 3.2. Energy Budget

To ensure the autarkic operation of the SiPB, the energy budget must be analyzed, considering both the energy demand depending on the measurement strategy and the energy that can be supplied through energy harvesting. In the following, the energy demand is first determined, before the required temperature difference for autarkic operation utilizing thermoelectrical EH is established.

#### 3.2.1. Energy Demand

The circuit board design of the SiPB discussed in this paper was not instrumented for power measurements of individual components. For this reason, the total power consumption was measured with a source measurement unit (SMU) for different software configurations. In each configuration, certain parts of the circuit were shut down to isolate their contributions. Measurements were taken at different sampling rates to further separate the static and dynamic power demands $P_{\mathrm{stat}}$ and $P_{\mathrm{dyn}}$. The SMU was connected directly to the GND (ground) and VDD (positive supply voltage) lines, effectively bypassing the DC-DC converter normally used to power the device [26]. This eliminates the energy losses introduced by the DC-DC converter. Additionally, a filter capacitor was utilized to average-out high-frequency variations in the dynamic load.

**Figure 17:**
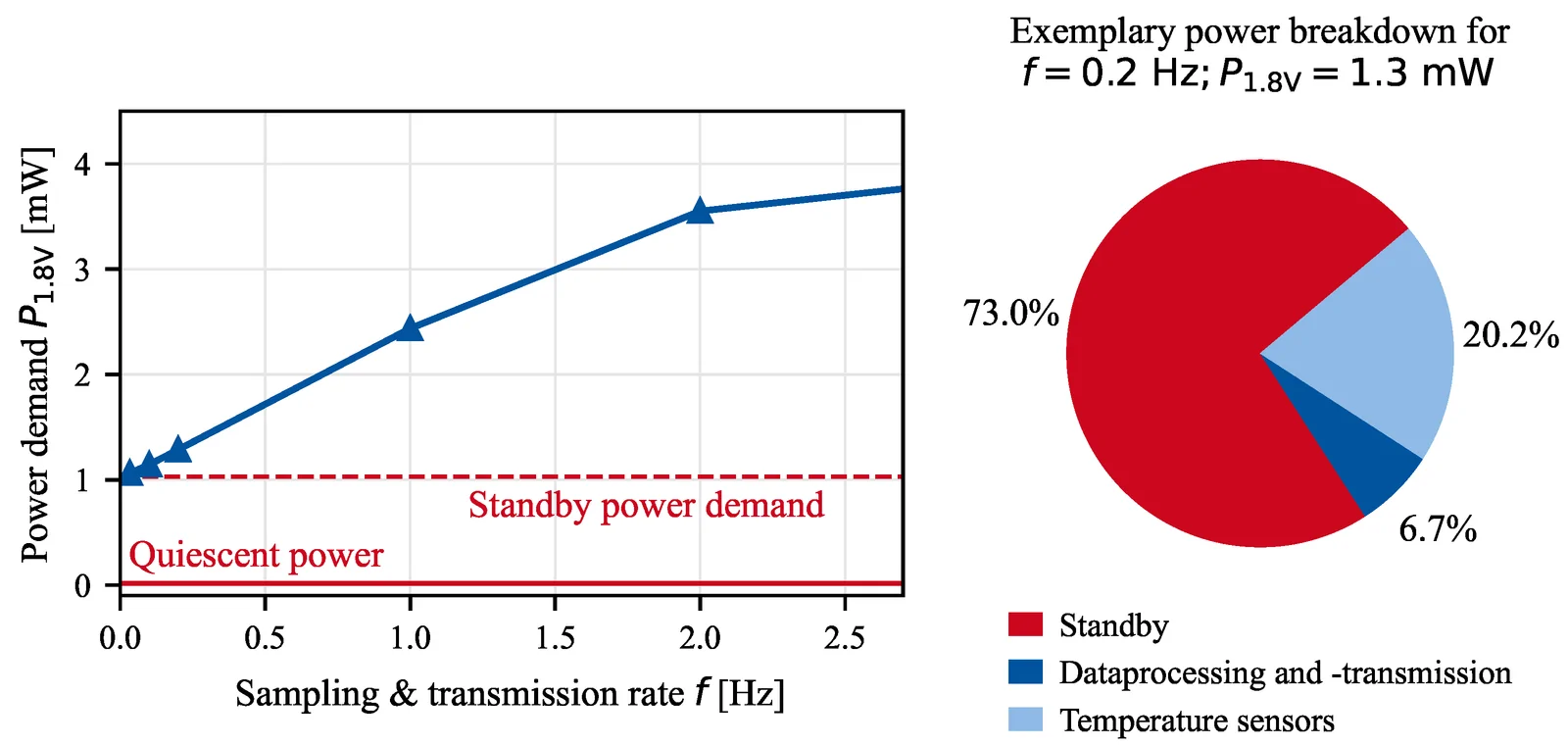
Sensor power demand as a function of sensor sampling rate.

The dashed red line in Figure 17 marks the standby power consumption of the nRF52805 [31], which constitutes the largest component of the static power consumption $P_{\mathrm{stat}}$. For low sampling rates, static power accounts for the majority of the total power consumption. At the transition to $P_{\mathrm{stat}}$-dominant behavior, Figure 17 provides a detailed breakdown of the contributors to the total power. In the current version of the PCB, the sleep clock is supplied with the same 64 MHz reference as the main clock. This explains the higher-than-usual static power consumption. A dedicated crystal oscillator or the internal RC oscillator is recommended to reduce the standby leakage power [31]. By utilizing a dedicated low-power real-time clock, the processor’s standby mode could be bypassed entirely by shutting down the processor completely and waking it up with an external alarm signal generated by the external real-time clock. Assuming that the power demand of the additional hardware is negligible, the theoretical maximum power reduction is given by the share of standby power consumption. At f=0.2Hz, the theoretical maximum power reduction is 73%. At f=0.033Hz, the theoretical maximum power reduction reaches 97%.

The dynamic power consumption is mainly caused by the sampling of the temperature sensors [30]. The blue curve in Figure 17 illustrates the relationship between the sampling frequency and power consumption. Up to 2 Hz, the power increases proportionally to the sampling rate. Around 2.5 Hz, the maximum sampling rate is reached with an approximate read-out time of 400 ms for all sensors. For sampling frequencies lower than 0.2 Hz, the power consumption approaches $P_{\mathrm{stat}}$ asymptotically.

A power breakdown similar to that in Figure 17 was established for the previous iteration of the prototype design. In the earlier version, the average power consumption was P=1.9mW at an operating frequency of f=0.2Hz [28]. In the current iteration, this power consumption was reduced by more than 30% to P=1.3mW by scaling down the supply voltage from 3.3V to 1.8V.

In the previous design, the sleep phase and the always-on components consumed 5.4mWs per measurement cycle. Based on the values presented in Figure 17, the energy consumption per cycle for the current iteration is calculated as E=0.73·1.3mW·5s=4.745mWs. This represents a modest reduction in energy consumption during inactive phases. Furthermore, the proportion of data processing was successfully reduced from 16.5% to 6.7%. In the previous version, WiFi was used due to ease of system integration. Moreover, the earlier wireless module [37] did not feature any benefits in power during transmissions using BLE. Its power consumption was not part of the reported power measurements during data capture in the measurements. The nRF52805, used in the current iteration, was specifically chosen for its ultra-low-power BLE functionality. The power breakdown in Figure 17 includes the overhead of wireless transmission in the data processing share.

#### 3.2.2. Laboratory Studies Thermoelectrical Energy Harvesting

**Figure 18:**
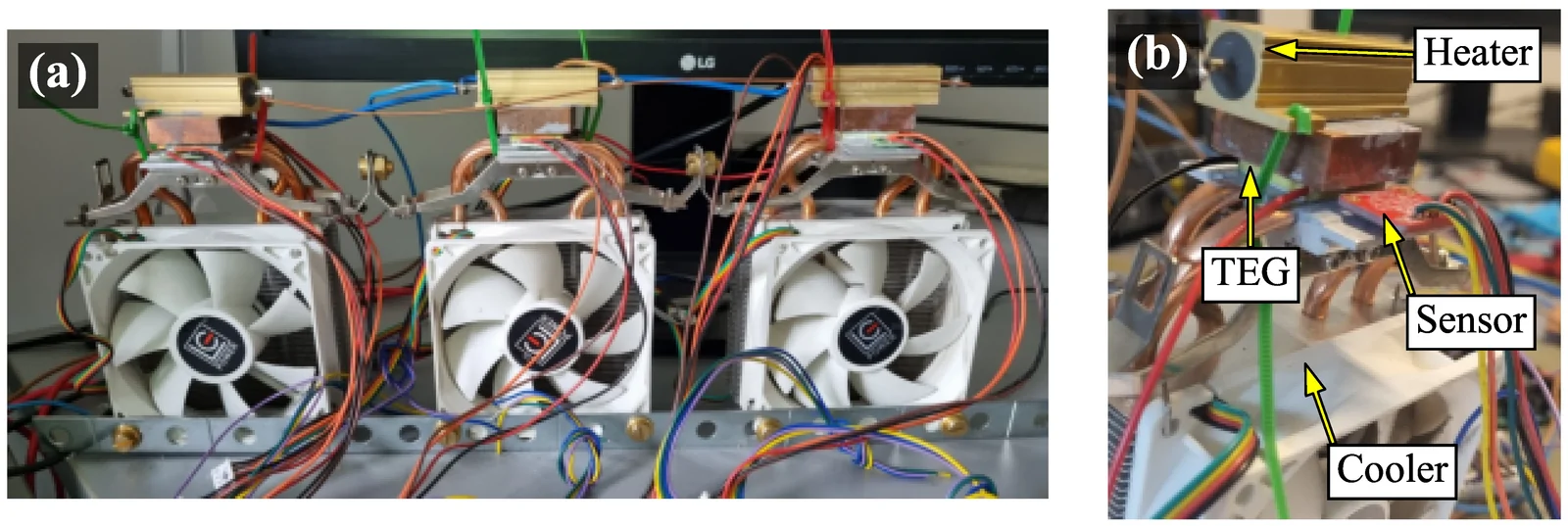
Laboratory TEG measurement setup. (**a**): Overall setup with three TEGs (**b**): Close-up of a TEG within the setup.

While previous publications on energy harvesting with the developed prototype relied primarily on simulation results, a dedicated test setup has now been developed to enable methodical measurement. In this measurement setup, which is displayed in Figure 18, three TEGs were connected in series to increase the total output voltage. Each TEG was subjected to a thermal gradient using a heating resistor on the upper surface and a fan for active cooling on the lower surface, as illustrated in Figure 18.

The resulting temperature differential (ΔT) was monitored via sensors positioned on both surfaces. As the electrical load, the prototype was connected and configured to operate at a low sampling rate (f=0.1Hz), resulting in maximum average power consumption of 1.1mW. The monitored variables included the TEG voltage and current, the storage capacitor voltage, and the current draw of the prototype. The supply voltage of the prototype was not actively measured, as it is dynamically regulated by the PMIC to a fixed value of 1.8V.

**Figure 19:**
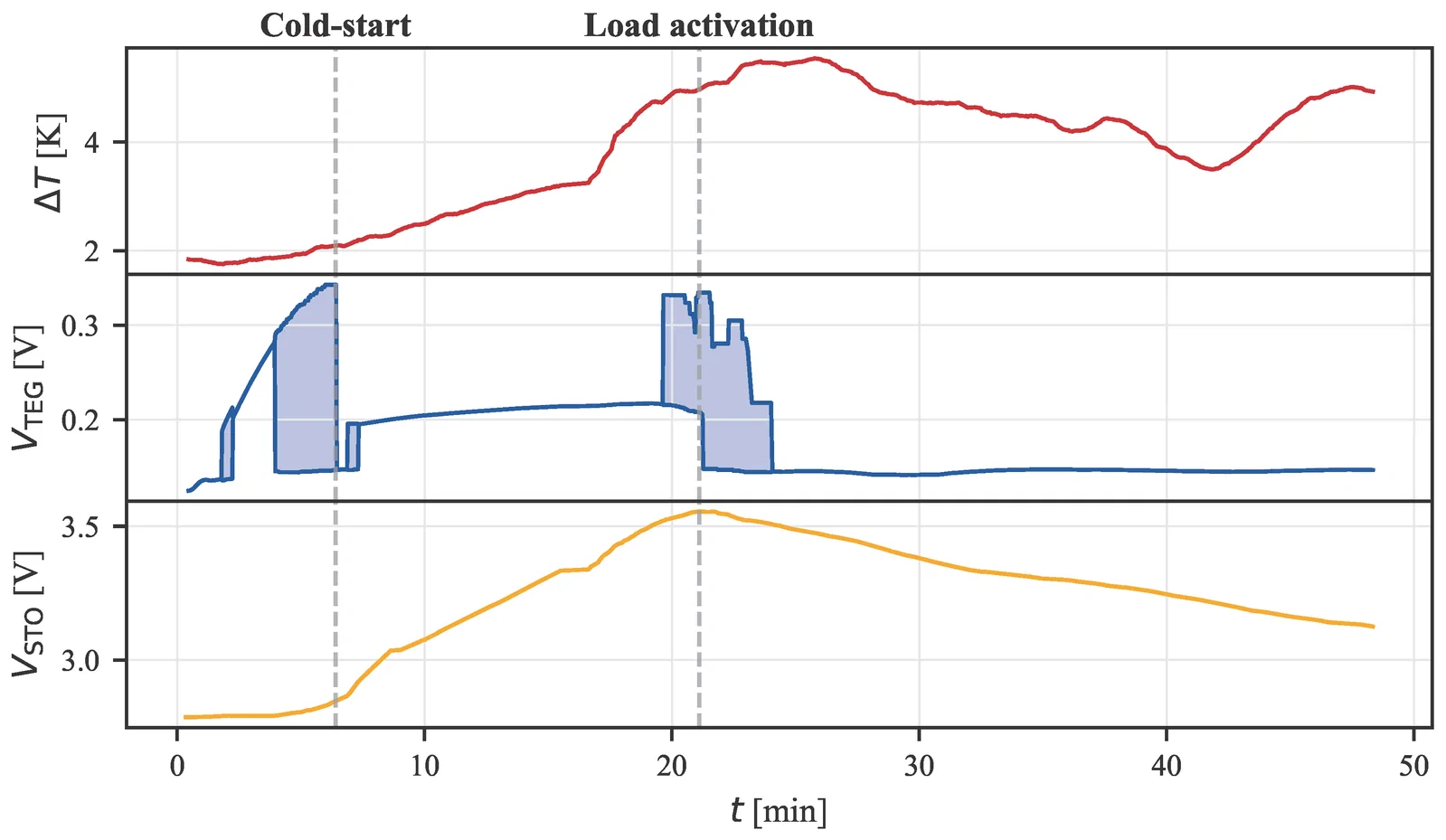
Thermoelectrical energy harvesting.

As shown in Figure 19, the blue curve represents the TEG output voltage ($V_{\mathrm{TEG}}$), where the shaded light blue regions highlight transient voltage spiking. This prominent spiking behavior occurs during power management integrated circuit (PMIC) mode transitions when a sudden change in load causes fluctuations in the voltage. The initial cold-start transition takes place at a temperature difference of approximately 2 K occurring at the TEGs. After surpassing this threshold, the storage capacitor voltage ($V_{\mathrm{STO}}$), represented by the orange curve, exhibits a sharp increase. Once the capacitor reaches the minimum voltage required by the PMIC, the PMIC transitions into supply mode [26], which coincides with the second spiking region in the graph. The temperature difference at the moment of activation was measured to be approximately 5 K. Subsequently, a significant drop in $V_{\mathrm{TEG}}$ is observed when the PMIC enables power output on its secondary side, followed by the gradual discharge of the storage capacitor. Previous simulation results for the deployed setup indicated a minimum expected temperature difference of 6 K for the front side where the TEGs are located [18]. Under these conditions, a cold start would be possible.

In this experimental setup, a thermal breakeven threshold, where the storage capacitor voltage stabilizes under a continuous load, was not achieved within the tested temperature range. Nevertheless, these measurements successfully captured the critical cold start and mode transition phases of the PMIC under low-temperature energy harvesting conditions. Investigating this transient startup phase provides valuable insights into the non-ideal behaviors and voltage dynamics that occur before reaching steady-state operation, which is essential for defining the minimum operational limits ($\Delta T_{\min}$) of the system.

## 4. Discussion

This paper presents the experimental investigation of temperature field measurement (TFM) as a promising approach for a developed sensor-integrating plain bearing (SiPB) prototype and determines the resulting energy demand with the aim of optimizing it. To this end, two particularly damage-critical abnormal events were experimentally investigated: overheating due to mechanical overload and particle contamination. A newly developed temperature field measurement (TFM) approach is used as the SiPB’s condition monitoring method. The TFM approach is characterized by its energy efficiency, enabling the SiPB to operate autarkically through energy harvesting methods. The measurement data are processed by a microcontroller embedded in the plain bearing. The measurement data and alarm signals are transmitted wirelessly via Bluetooth Low Energy.

Two tests were conducted for the abnormal event of overheating, while only one test was conducted for particle contamination. Based on these single tests, TFM-based characteristics were identified that indicate the potential for the early detection of damage. In the tests performed, characteristic changes in the TFM signal occurred before the abnormal events became damage-critical. These characteristics were derived descriptively (Table 5) and are not validated alarm thresholds. Differentiation between the abnormal events, and between abnormal events and regular operation under varying loads, sliding speeds, and oil temperatures, cannot be achieved from the present data and is a subject for future work. Mechanical overload was characterized by an initial local temperature rise ($\Delta T_{local}$), followed by a large global temperature gradient ($\Delta T_{global}$) accompanied by a high temperature level ($T_{max}$). Particle contamination, in contrast, was characterized by an instantaneous local temperature increase ($\Delta T_{local}$) upon particle ingress into the bearing, followed by an increased interquartile range (IQR) of the mean temperature over time, with the temperature level additionally correlating strongly with the friction torque over the course of the event.

For mechanical overload, a high temperature level and high local temperature gradients were detected before the onset of damage. Discontinuities in the temperature field signal were detected at least 10 min before overheating damage occurred in both overheating tests. These discontinuities begin at high φ-angle positions and low temperature levels and gradually migrate over time to lower φ-angle positions and higher temperature levels. To further investigate the underlying physical mechanisms causing these temperature discontinuities, repeating the overheating tests while simultaneously measuring the thermoelectric contact voltage using the Bearomos sensor [38] is planned. By measuring the contact voltage, it will be possible to determine whether the signal discontinuities are caused by localized mixed friction, thereby identifying the underlying mechanisms that initiate the damage.

For particle contamination, the single test showed that even very light friction torque increases caused by three-body abrasion were accompanied by a considerably more erratic temperature distribution. The IQR of the temporal distribution of the average temperature is a promising characteristic for indicating increased particle contamination, but its general applicability and a threshold value still have to be validated via repeated tests: in the contaminated test, the IQR was approximately 2.6 times higher than in the running-in test, and this erratic behavior persisted throughout the entire time course. Furthermore, the temperature distribution correlates very strongly with the friction torque curve.

These initial experiments have shown that the temperature differences required for a cold start match previous simulation results for temperature differences achievable inside the bearing. The conditions for the continuous operation of the SiPB remain unvalidated. Further research targeting PMIC power delivery is therefore required to establish concrete energy optimization goals. Further experimental validation is needed to assess whether the necessary temperature differences are reached for a wide range of speeds and loads within the typical ranges of common real-world applications. To also support less favorable conditions, the power consumption should be further reduced. For this, the next iteration of the setup will optimize the sleep-mode power consumption with a dedicated low-power sleep clock. Furthermore, the energy harvesting efficiency will be optimized using the integrated maximum power point tracking (MPPT) of the AEM13920 [26].

## 5. Conclusions

A temperature field-based condition monitoring approach (TFM-based CMS) was experimentally investigated for the early detection of two abnormal events in hydrodynamic plain bearings: particle contamination and overheating due to mechanical overload. A developed sensor-integrating plain bearing (SiPB) prototype was used for the experimental investigation (inner diameter: $d_{i}=120$ mm). In addition, the energy demand of the prototype and the feasibility of thermoelectric energy harvesting for the autarkic operation of the SiPB were investigated. The main findings are as follows.

- Overheating due to mechanical overload developed through a high temperature level ($T_{max}$) and a global temperature gradient $\Delta T_{global}$ ($=T_{max}-T_{min}$) that increased over time, reaching up to 25.6 °C at shutdown. In both overheating tests, characteristic discontinuities in the temperature field signal occurred at least 10 min before failure due to exceeding the friction torque limit.
- Particle contamination led to an immediate local temperature increase due to three-body abrasion of approximately $\Delta T_{local}=4$ °C and to a threshold increase in the interquartile range (IQR) of the mean temperature (0.18 °C compared to 0.07 °C in the running-in test) by a factor of 2.6. Both effects occurred in a state of very light mixed friction ($M_{R}$ from 2 Nm to approximately 6 Nm) and correlated with the friction torque.
- The energy demand of the SiPB prototype amounts to 1.3 mW at a sampling rate of 0.2 Hz. In order to ensure a constant operating voltage for the SiPB’s microelectronic components, the voltage generated thermoelectrically must be converted using a DC-DC converter. The cold start and the transition to supply mode of the DC-DC converter were demonstrated at temperature differences of approximately 2 K and 5 K, respectively. The thermal breakeven point under a continuous load was not reached within the tested temperature range.

The results indicate that a simple approach with a sampling rate in the Hz range may be sufficient to detect the investigated abnormal events in a timely manner. The presented single-trial investigations indicate the feasibility of the SiPB’s energy autarky potential by thermoelectric energy harvesting within the machine element; the results are to be understood as a proof of concept for the investigated bearing setup.

Table 5 consolidates the TFM-based characteristics for each failure mode together with the conditions under which they were derived and the lead time before the friction torque limit $M_{R,limit}$ = 450 Nm was exceeded.

The results presented are valid for the investigated bearing setup (bearing size and material, test rig). Each test investigating the abnormal events of overheating due to mechanical overload and particle contamination was only conducted once and is presented for the first time in this paper.

The temperature sensors cover only a limited angular sector of the bearing (180°–266.25°). This sector was chosen based on the displacement angle under steady-state conditions, which does not cover abnormal events. In both overheating tests, $\varphi_{T,max}$ shifted to 187.5° shortly before the friction torque limit was exceeded, only 7.5° from the edge of the sensor array. It therefore cannot be excluded that the true temperature maximum was located outside the instrumented sector in this phase. The $T_{max}$ and $\Delta T_{global}$ measured by the SiPB then represent lower bounds on the actual values, the severity of the event may be underestimated, and $\varphi_{T,max}$ saturates at the array boundary. Detection itself is not impaired, since the temperatures rose at all measurement positions before the friction torque limit was reached. Future work will extend the angular coverage of the temperature field-based condition monitoring approach, e.g., with a larger or adaptive sensor sector.

The reported characteristics were derived descriptively and are not statistically validated thresholds. The objective of this paper was therefore to indicate, for the first time, the potential for early damage detection for the two abnormal events using the SiPB. TFM-based characteristics were identified that develop for these abnormal events prior to an increase in friction torque. Repeated testing is required to assess repeatability and to determine false alarm and missed detection rates. Subsequently, the differentiation of abnormal events and damage causes from one another and from regular operation (varying load, sliding speed, and oil temperature) will be investigated. In addition, the energy demand will be reduced further, e.g., through a low-power sleep clock and reduced use of the temperature sensors, to prove the permanent autarky of the SiPB. Finally, a follow-up transfer project is planned, where the concept will be transferred from the test rig to field applications.

## Figures and Tables

**Table 1:** Key specifications of the integrated sensor system, power management components, and energy harvesters of the SiPB prototype.

Component/Domain	Parameter	Value/Specification
Temperature Sensors	Component	TI TMP117
	Quantity	24
	Angular resolution	$3.75^{\circ }$
	Angular coverage	$180^{\circ }$–$266.25^{\circ }$
	Max. sampling rate	8.06Hz
	Max. operating temperature ($T_{\max}$)	150 °C
	Temperature resolution (ΔT)	0.0078 °C
Energy Harvesters	Component	MGM250-71-10-16 TEG
	Quantity	3
	Edge length	22mm
Energy Storage	Component	Gold-Cap (Capacitor)
	Capacitance (*C*)	0.47F

**Table 2:** Overview of test rig configuration.

	GLP120
Bearing inner diameter $d_{i}$ [mm]	120
Rel. bearing clearance ψ [-]	1.1 ‰
Bearing material	CuSn12Ni2-C
Bearing roughness Ra [μm]	0.5
Shaft material	42CrMo4
Shaft roughness Ra [μm]	0.5
Particle type	Iron dust 4–6 μm
Particle concentration (Section 3.1.2) $c_{Particles}$ [-]	0.1 g/100 g

**Table 3:** Selected TFM-based parameters and friction torque values for the test section shown in Figure 8.

*t* [Min]	$T_{\min}$ [°C]	$T_{\max}$ [°C]	$\Delta T_{\mathrm{global}}$ [°C]	$M_{R}$ [Nm]	$\varphi_{T,\max}$ [°]
277.50	71.2	75.4	4.2	4.635	225.00
280.00	71.9	76.0	4.1	2.151	225.00
282.50	71.3	77.7	6.4	19.261	232.50
285.00	72.6	79.8	7.2	30.197	236.25
287.50	74.5	82.9	8.5	53.602	213.75
289.69	94.1	119.7	25.6	534.614	187.50

**Table 4:** Selected TFM-based parameters and friction torque values for the test section shown in Figure 10.

*t* [Min]	$T_{\min}$ [°C]	$T_{\max}$ [°C]	$\Delta T_{\mathrm{global}}$ [°C]	$M_{R}$ [Nm]	$\varphi_{T,\max}$ [°]
4.40	87.6	95.8	8.1	3.873	213.75
10.00	91.4	103.9	12.5	2.668	213.75
15.00	93.1	106.4	13.3	3.093	213.75
20.00	97.1	110.9	13.7	4.419	213.75
22.00	100.8	114.2	13.4	3.107	195.00
24.12	109.8	124.8	15.0	482.438	187.50

**Table 5:** Consolidated TFM-based alarm characteristics per failure mode, their derivation conditions, and the lead time before $M_{R,limit}$.

Failure Mode	Parameter	Value	Derivation Conditions ($\bar{p}$/$\bar{v}$/$T_{\mathrm{Initial}}$/Sampling Rate)	Lead Time Before $M_{R,\mathrm{limit}}$
Overload/overheating, test 1 (two pressure steps)	Discontinuities in the temperature field signal (Figure 12)	Duration Δt = 3–6 min at a fixed angular position φ	30 MPa → 40 MPa (load step at *t* = 240 min)/1.5 m/s/60 °C/ 2.5 Hz	≥10 min
	$\Delta T_{local}$ at load increase	6.8 °C within 7.5 min (φ = 225.00°, *t* = 280–287.5 min)		2.19 min
	$\Delta T_{global}$	3.0 °C → 4.0 °C at load step; 6.4 °C at onset of mixed friction (*t* = 282.5 min); 25.6 °C at shutdown		Approximately 7 min (at 6.4 °C)
Overload/overheating, test 2 (single-step)	Discontinuities in the temperature field signal (Figure 12)	Duration Δt = 3–6 min at a fixed angular position φ	60 MPa/3 m/s/80 °C/2.5 Hz	≥10 min
	$\Delta T_{global}$	>10 °C (12.5 °C at *t* = 10 min), approximately constant until shutdown		Approximately 14 min
	$T_{max}$	Continuous rise up to 124.8 °C at shutdown		4.87 min ($T_{max}$ > 110 °C)
Particle contamination	Interquartile range (IQR) of the mean temperature	0.18 °C vs. 0.07 °C in regular operation	20 MPa/0.5 m/s/60 °C/2.5 Hz; 0.1 g/100 g iron dust, $d_{Particle}$ = 4–6 µm	$M_{R,limit}$ not reached; detectable immediately after contamination
	$\Delta T_{local}$	Approximately 4 °C at all positions		Immediately after contamination

Values were derived descriptively from one test per operating condition and are not statistically validated alarm thresholds.

## Data Availability

The data presented in this study are available on request from the corresponding author.

## Abbreviations

The following symbols and abbreviations are used in this manuscript:

BLE	Bluetooth Low Energy
CMS	Condition monitoring system
GLP120	120 mm plain bearing test rig
GND	Ground
IQR	Interquartile range
MPPT	Maximum power point tracking
PAO	Polyalphaolefin
PMIC	Power management integrated circuit
SiME	Sensor-integrating machine elements
SiPB	Sensor-integrating plain bearing
SMU	Source measurement unit
TEG	Thermoelectrical generator
TFM	Temperature field measurement
VDD	Positive supply voltage

## References

[1] (2017). Gleitlager–Hydrodynamische Radial-Gleitlager im stationären Betrieb: Teil 3: Betriebsrichtwerte für die Berechnung von Kreiszylinderlagern.

[2] (2017). Gleitlager–Hydrodynamische Radial-Gleitlager im stationären Betrieb: Teil 2: Funktionen für die Berechnung von Kreiszylinderlagern.

[3] (2017). Gleitlager–Hydrodynamische Radial-Gleitlager im stationären Betrieb: Teil 1: Berechnung von Kreiszylinderlagern.

[4] König F., Sous C., Jacobs G. (2021). Numerical prediction of the frictional losses in sliding bearings during start-stop operation. Friction.

[5] Lucassen M., Decker T., Guzmán F.G., Lehmann B., Bosse D., Jacobs G. (2023). Simulation methodology for the identification of critical operating conditions of planetary journal bearings in wind turbines. Forsch. Ingenieurwesen.

[6] Sander D.E., Allmaier H., Priebsch H.H., Darji P.H. (2016). Friction and Wear in Automotive Journal Bearings Operating in Today’s Severe Conditions. Advances in Tribology.

[7] König F. (2020). Prognose des Verschleißverhaltens Ölgeschmierter Gleitlager im Mischreibungsbetrieb. Ph.D. Thesis.

[8] Sander D.E., Allmaier H. (2018). Starting and stopping behavior of worn journal bearings. Tribol. Int..

[9] Prölß M., Schwarze H., Hagemann T., Zemella P., Winking P. (2018). Theoretical and Experimental Investigations on Transient Run-Up Procedures of Journal Bearings Including Mixed Friction Conditions. Lubricants.

[10] Damm K., Pucher K., Skiadas A., Witt M. (2015). Sputter Bearings for Highly Charged Diesel Engines. MTZ Worldw..

[11] König F., Wirsing F., Jacobs G., He R., Tian Z., Zuo M.J. (2023). Bayesian inference-based wear prediction method for plain bearings under stationary mixed-friction conditions. Friction.

[12] Boucherit H., Bou-Saïd B., Lahmar M. (2017). The effect solid particle lubricant contamination on the dynamic behavior of compliant journal bearings. Lubr. Sci..

[13] Poddar S., Tandon N. (2019). Detection of particle contamination in journal bearing using acoustic emission and vibration monitoring techniques. Tribol. Int..

[14] Mokhtari Molk Abadi N. (2020). Überwachung Hydrodynamischer Gleitlager Basierend auf der Körperschallanalyse. Ph.D. Thesis.

[15] Ma J., Yu J., Zhou X., Gu F., Jiang L., Li X. (2026). Condition monitoring of journal bearings based on acoustic emissions: A state-of-the-art review. Friction.

[16] Wu L., Palamarciuc I., Bajwa R., Zhang Y., Dwyer-Joyce R. (2026). An in-situ wear measurement instrumentation for thin metallic bearing coatings using high frequency ultrasound. Tribol. Int..

[17] Kirchner E., Wallmersperger T., Gwosch T., Menning J.D.M., Peters J., Breimann R., Kraus B., Welzbacher P., Küchenhof J., Krause D. (2024). A Review on Sensor–Integrating Machine Elements. Adv. Sens. Res..

[18] Baszenski T., Kauth K., Kratz K.H., Gutiérrez Guzmán F., Jacobs G., Gemmeke T. (2023). Sensor integrating plain bearings: Design of an energy-autonomous, temperature-based condition monitoring system. Forsch. Ingenieurwesen.

[19] Nejad A.R., Purcell E., Valavi M., Hudak R., Lehmann B., Gutiérrez Guzmán F., Behrendt F., Bõhm A.M., von Bock und Polach F., Nickerson B.M. (2021). Condition Monitoring of Ship Propulsion Systems: State-of-the-Art, Development Trend and Role of Digital Twin. Proceedings of the Polar and Arctic Sciences and Technology, Virtual, 21–30 June 2021.

[20] Laubichler C., Kiesling C., Da Marques Silva M., Wimmer A., Hager G. (2022). Data-Driven Sliding Bearing Temperature Model for Condition Monitoring in Internal Combustion Engines. Lubricants.

[21] Aufischer R., Esmael B., Hager G., Hamdard K., Schallmeiner S., Breiteneder T., Taucher G., Vystejn J., Wimmer A., Geringer B., Lenz H.P. (2019). Intelligente Gleitlager in der Motorenentwicklung und Validierung/Intelligent Bearings for Engine Development and Validation. Proceedings of the 40 Internationales Wiener Motorensymposium, Vienna, Austria, 15–17 May 2019.

[22] Baszenski T., Groß J., Kratz K.H., Paeßens J., Jacobs G., Gemmeke T., Lehmann B., Lucassen M. (2025). Experimental and simulative investigation of an energy-autarkic, temperature field-based Sensor-integrating Plain Bearing focusing transient operating conditions. Forsch. Ingenieurwesen.

[23] (1996). Gleitlager–Teil 2: Temperaturüberwachung.

[24] Rowe D.M. (2005). Thermoelectrics Handbook: Macro to Nano.

[25] Bancik K., Konecny J., Stankus M., Hercik R., Koziorek J., Markevičius V., Andriukaitis D., Prauzek M. (2026). Batteryless IoT Sensing Using Thermoelectric Energy Harvesting from Industrial Motor Waste Heat. Sensors.

[26] e-peas S.A. (2026). Ultra Efficient Dual Sources Energy Manager with Ratio/Constant Voltage Regulation, Regulated Buck Output and 5 V Charger. Version 2.5, Document DS-AEM13920-v2.5. https://e-peas.com/documents/AEM13920/DS-AEM13920.pdf.

[27] Kirchner E., Martin G., Vogel S. Sensor integrating machine elements—Key to in-situ measurements in mechanical engineering. Proceedings of the 23rd International Seminar on High Technology.

[28] Paeßens J., Kratz K.H., Gemmeke T., Kauth K., Baszenski T., Lehmann B., Jacobs G. (2024). Design of a fully integrated sensor system of a plain bearing. Forsch. Ingenieurwesen.

[29] European Thermodynamics (2022). MGM250-71-10-16 Mini Thermoelectric Generator Module. European Thermodynamics Ltd. (Adaptive), Version 1. https://www.adaptivete.com/wp-content/uploads/2022/10/MGM250-71-10-16.pdf.

[30] Texas Instruments (2022). TMP117 High-Accuracy, Low-Power, Digital Temperature Sensor With SMBus™- and I2C-Compatible Interface. Document SNOSD82D. https://www.ti.com/lit/ds/symlink/tmp117.pdf.

[31] Nordic Semiconductor (2023). nRF52805 Product Specification. Version 1.4. https://docs.nordicsemi.com/r/bundle/ps_nrf52805/page/keyfeatures_html5.html.

[32] Castrol (2017). Data sheet: Castrol Optigear Synthetic CT 320. https://msdspds.castrol.com/bpglis/FusionPDS.nsf/Files/DEA5BA71A44DA288802581E9003D5BEA/$File/BPXE-AU2JDS.pdf.

[33] Decker T., Jacobs G., Raddatz M., Röder J., Betscher J., Arneth P. (2025). Detection of particle contamination and lubrication outage in journal bearings in wind turbine gearboxes using surface acoustic wave measurements and machine learning. Forsch. Ingenieurwesen.

[34] CJC (2026). Condition Monitoring Unit—Oil Condition Sensors. Karberg & Hennemann GmbH & Co. KG, PDMO4009-1-UK. https://www.cjc.de/en/condition-monitoring-unit/.

[35] Cleff T. (2015). Deskriptive Statistik und Explorative Datenanalyse.

[36] (2008). Gleitlager–Erscheinungsbild und Charakterisierung von Schäden an Ölgeschmierten Metallischen Gleitlagern: Teil 1: Allgemeines (ISO 7146-1:2008).

[37] Espressif Systems (2026). ESP32-C3 Series Datasheet. Version 2.4. https://documentation.espressif.com/esp32-c3_datasheet_en.pdf.

[38] Schaller Automation (2020). Bearomos 2020–Bearing Monitoring System for Oil-Lubricated Slide Bearings. SCHALLER Automation, BEAROMOS V3.01. https://schaller-automation.com/wp-content/uploads/2023/05/Brochure_Bearomos_EN_05-23_LOW-1.pdf.

